# Response of chickpea (*Cicer arietinum* L.) to terminal drought: leaf stomatal conductance, pod abscisic acid concentration, and seed set

**DOI:** 10.1093/jxb/erw153

**Published:** 2016-04-20

**Authors:** Jiayin Pang, Neil C. Turner, Tanveer Khan, Yan-Lei Du, Jun-Lan Xiong, Timothy D. Colmer, Rosangela Devilla, Katia Stefanova, Kadambot H.M. Siddique

**Affiliations:** 1 School of Plant Biology, The University of Western Australia, M084, LB 5005 Perth, WA 6001, Australia; 2 The UWA Institute of Agriculture, The University of Western Australia, M082, LB 5005 Perth, WA 6001, Australia; 3 State Key Laboratory of Grassland Agroecosystems, Institute of Arid Agroecology, School of Life Sciences, Lanzhou University, Lanzhou 730000, Gansu Province, China; 4 CSIRO Agriculture, Black Mountain Laboratories, Canberra, ACT 2601, Australia

**Keywords:** Abscisic acid, flower abortion, fraction of transpirable soil water, photosynthesis, pod abortion, pollen viability and germination, seed filling, water deficit.

## Abstract

Flower and pod production and seed set of chickpea (*Cicer arietinum* L.) are sensitive to drought stress. A 2-fold range in seed yield was found among a large number of chickpea genotypes grown at three dryland field sites in south-western Australia. Leaf water potential, photosynthetic characteristics, and reproductive development of two chickpea genotypes with contrasting yields in the field were compared when subjected to terminal drought in 106kg containers of soil in a glasshouse. The terminal drought imposed from early podding reduced biomass, reproductive growth, harvest index, and seed yield of both genotypes. Terminal drought at least doubled the percentage of flower abortion, pod abscission, and number of empty pods. Pollen viability and germination decreased when the fraction of transpirable soil water (FTSW) decreased below 0.18 (82% of the plant-available soil water had been transpired); however, at least one pollen tube in each flower reached the ovary. The young pods which developed from flowers produced when the FTSW was 0.50 had viable embryos, but contained higher abscisic acid (ABA) concentrations than those of the well-watered plants; all pods ultimately aborted in the drought treatment. Cessation of seed set at the same soil water content at which stomata began to close and ABA increased strongly suggested a role for ABA signalling in the failure to set seed either directly through abscission of developing pods or seeds or indirectly through the reduction of photosynthesis and assimilate supply to the seeds.

## Highlight

Exposed to terminal drought, the soil water content at which chickpea seed set ceased coincided with that at which stomatal conductance began to decrease and pod abscisic acid concentration increased.

## Introduction

Chickpea (*Cicer arietinum* L.) is the second most important grain legume (pulse) globally, occupying 13.5 Mha, and is the largest pulse crop in Australia, currently grown on >0.5 Mha (FAOSTAT, 2013; http://faostat3.fao.org/faostat-gateway/go/to/download/Q/QC/E). In subtropical areas (South Asia, eastern Africa, north-eastern Australia), it is grown on stored soil moisture after the rainy season; while in Canada and Mediterranean climatic regions (e.g. the Mediterranean basin and southern Australia) it is grown in the rainy season (Leport *et al.*, 1999). Whether grown on stored soil moisture or current rainfall which declines during autumn, chickpea is exposed to water shortage during the reproductive phase, a situation referred to as ‘terminal drought’ (Siddique *et al.*, 1999).

Previous studies have shown that during terminal drought, chickpea seed yield decreases significantly compared with irrigated plants, due to flower and pod abortion, reduced pod production, and reduced seed size (Leport *et al.*, 1998, 1999; Davies *et al.*, 1999; Fang *et al.*, 2010). Fang *et al.* (2010) found that drought stress impaired not only pollen viability but also stigma/style function. However, it is not clear whether the flower and pod abortion was due to failure of the pollen tube to reach the ovary or other factors such as lack of carbohydrates and/or hormonal interactions. Studies have shown that drought-induced abscisic acid (ABA) concentration in developing floral organs is related to increased abortion of reproductive sinks in maize (*Zea mays* L.) (Ober *et al.*, 1991), wheat (*Triticum aestivum* L.) (Westgate *et al.*, 1996), and soybean (*Glycine max* L.) (Liu *et al.*, 2003). The role of ABA in flower and pod abortion in chickpea subjected to terminal drought has not been evaluated.

Physiological processes of plants in drying soil do not begin to decrease immediately after water is withheld, but there is a threshold soil water content at which transpiration and other physiological processes begin to decrease (Zaman-Allah *et al.*, 2011; Pushpavalli *et al.*, 2015). In chickpea, studies on the threshold values when transpiration begins to decrease show large genotypic variation when drought stress was imposed during either the vegetative or the reproductive stage (Zaman-Allah *et al.*, 2011; Pushpavalli *et al.*, 2015). In grass pea (*Lathyrus sativus* L.), the threshold soil water content at which seed set was reduced coincided with that at which the leaf stomatal conductance and photosynthetic rate began to decrease (Kong *et al.*, 2015). To date, no studies on the threshold values of soil water content at which the development of reproductive organs (flowers, pods, and seeds) ceases have been undertaken in chickpea, and their association with plant water status, photosynthetic characteristics, and phytohormone production is unknown.

Here we report on the seed yield of >100 chickpea genotypes grown over 2 years at three dryland field sites in south-western Australia, and a detailed glasshouse experiment on the physiological and yield responses to terminal water deficit of two genotypes with contrasting field yields. The objective of the field experiments was to assess the genetic variation in yield of chickpea genotypes, including Indian-derived and Australian breeding lines, under typical semi-arid Mediterranean-type climatic conditions of the grain belt of south-western Australia in which terminal drought frequently occurs. Due to variation in flowering time, leaf area, rates of transpiration, and root growth, different genotypes often experience different degrees of water deficit in the field, particularly during reproductive growth. To investigate whether the observed yield differences in the field were due to genotypic differences in sensitivity to terminal drought during the reproductive phase, two genotypes with similar phenology and showing yield differences in the field were exposed to a slow, controlled, progressive water deficit in large soil containers in a glasshouse to ensure a similar rate of soil drying in both genotypes, an approach similar to that used in other studies (Zaman-Allah *et al.*, 2011; Pushpavalli *et al.*, 2015). The objectives of the glasshouse study were to investigate for two chickpea genotypes: (i) the effects of terminal drought on reproduction, including flower and pod production and abortion, seed set, seed size, and seed yield; (ii) the threshold values of the fraction of transpirable soil water (FTSW) for cessation of flower, pod, and seed production, and for a reduction in leaf water potential, stomatal conductance, transpiration, and photosynthesis; and (iii) whether flower/pod abortion was due to the inhibition of pollen germination and pollen tube growth, or associated with increases in ABA during the development of the pod wall and seed under terminal drought.

## Materials and methods

### Field experiments

The field experiments were conducted over the winter–spring growing season (May–November) at one site in 2012 and two sites in 2013 in the grain belt of south-western Australia. In 2012, 108 chickpea genotypes (predominantly desi type) specifically selected for yield potential and adaptation to water-limited environments, as well as current check cultivars, were sown at York, Western Australia. The parentage of these lines was made up of Indian-derived and Australian breeding lines. In 2013, 62 genotypes including 50 breeding lines and 12 commercial cultivars (57 desi type and five Kabuli type) were grown at Bindi Bindi and at Cunderdin, Western Australia. Daily rainfall, and minimum and maximum temperatures during the growing season at the three sites are presented in Supplementary Fig. S1 at *JXB* online. The experiments received 230, 197, and 196mm of rainfall over the growing season at York in 2012, and at Bindi Bindi and Cunderdin in 2013, respectively, and no irrigation was supplied. Detailed information on the agronomic practices is given in the legend of Supplementary Fig. S2. Seeds from the whole plots were machine harvested at physiological maturity and the seed yield from each plot was measured after seeds were oven-dried at 30 °C for 7 d.

### Glasshouse experiment

#### Plant material and growth conditions

Two desi chickpea breeding lines with similar phenology, DICC8156 and DICC8172, were selected based on the field experiments in 2012 and 2013, with DICC8156 having consistently lower yield and DICC8172 having consistently higher yield across the three sites. The two genotypes had the same pedigree (ICCV96836/PBG5). The experiment was conducted from May to November 2014 in a temperature-controlled glasshouse at the University of Western Australia, Perth, Australia (31.57°S, 115.47°E) with an average maximum air temperature of 23 °C, a minimum temperature of 13 °C, and a mean relative humidity of 59%.

Plants were grown in large (80 litre) containers 460 mm×470mm at the top, 290 mm×190mm at the bottom, and a height of 770mm (Sulo, Somersby, NSW, Australia). The bottom of each container had five 12mm diameter drainage holes. Before planting, 5kg of coarse gravel was placed at the bottom of each container and covered by a piece of nylon mesh, and 105.6kg of a 4:1 mixture of sieved, dried loamy soil and river sand was added above the gravel to a soil depth of 630mm. The soil was collected from the upper 0.15 m layer of soil at the site of the 2013 field experiment at Cunderdin (31.64°S, 117.24°E). The soil is a reddish-brown sandy clay loam (clay=27%, silt=9%, sand=64%), classified as Red Calcic Dermosal (Isbell, 1996). The field soil contained 6 μg g^−1^ of nitrate-N, 3 μg g^−1^ of ammonium-N, 46 μg g^−1^ of Colwell P, 691 μg g^−1^ of Colwell K, and had a pH (CaCl_2_) of 7.1. After filling, the soil in the containers had a bulk density of 1.60g cm^−3^. Prior to filling the containers, diammonium phosphate (18% N and 20% P) at a rate of 0.016 μg g^−1^ soil was mixed well with the soil.

Two days prior to sowing, all containers were watered to 80% of field (i.e. pot) capacity (FC). The water content at FC was 19% (w/w). The soil water content at 100% FC was pre-determined by inundating 6kg replicates of dry soil with water in free draining pots, allowing it to drain for 48h, and then taking measurements of subsamples before and after oven-drying at 105 ^o^C. A custom-made balance, with a maximum capacity of 200kg and an accuracy of 10g, was used to weigh the containers to monitor soil water contents. At 80% FC, the soil mixture in each container contained 16 litres of water.

On 22 May 2014, 15 seeds were planted in each container at ~25mm and thinned to five plants at 18 days after sowing (DAS). At planting, the soil in each container was inoculated with ~10g of peat-based Group N rhizobium (New Edge Microbials, Albury, NSW, Australia). Immediately after the seedlings were thinned, the soil surface of each container was covered with 1.2kg of plastic beads (~30mm thick) to minimize soil evaporation.

Twelve containers were used for each genotype. The experiment had two water treatments and three replicates, with each replicate per treatment per genotype composed of two containers which were placed next to each other. One plant in each container was used for tagging all flowers and pods and for final harvest, while the remaining four plants in each container were used for destructive measurements such as leaf water potential, pollen germination, pollen viability and pollen tube growth, seed growth, and ABA concentration of pods and seeds, as well as non-destructive measurements of gas exchange. All containers were watered to 80% FC by weighing every 2 d until the two watering treatments were imposed.

#### Watering treatments

The two watering treatments were imposed at 100 DAS when all plants were at the early podding stage: (i) six containers of each genotype were kept well watered by daily watering (WW); and (ii) another six containers were exposed to water stress (WS). After the start of the two water treatments, all containers were weighed daily at 17:30h [Australian Western Standard Time (WST)]. The WW plants were maintained at 80% FC by daily watering until the WS plants reached maturity (144 DAS). The WS treatment was imposed gradually by rewatering the containers (Zaman-Allah *et al.*, 2011; Pushpavalli *et al.*, 2015) so that a maximum of 750ml of soil water from each container was lost each day until daily water loss was below 750ml. This was to avoid a too rapid imposition of water stress and to reset the soil water content at the same level regardless of plant size and water use (Zaman-Allah *et al.*, 2011; Pushpavalli *et al.*, 2015). A total of 7.15kg and 6.84kg of water was added to each container for the WS containers of DICC8156 and DICC8172, respectively, during the first 14 d of treatment. Soil water content at different depths was monitored using a Diviner2000 portable soil water monitoring probe (Sentek Sensor Technologies, Stepney, SA, Australia), via a 1.0 m high, 0.05 m diameter vertical access tube installed in the middle of each container (Pang *et al.*, 2013). The results from the Diviner2000 were calibrated with gravimetric measurements of soil water content in 1 m pots (15mm in diameter) filled with the same soil as that in the experiment (*R*^2^=0.99).

#### Estimation of the fraction of transpirable soil water

The FTSW values represent the fraction of the remaining soil water available for transpiration on each day of the experiment. The difference in container weight when plants were watered to 80% FC prior to the start of the water stress and that when transpiration had become negligible (no water available for transpiration) is the total transpirable soil water (TTSW) of the containers (Pushpavalli *et al.*, 2015). Daily FTSW=(daily container weight–final container weight)/(initial container weight–final container weight) (Ray and Sinclair, 1998). FTSW was back-calculated for each day of the experiment at the end of the experiment. The FTSW values are presented between 1 (at 80% FC) and 0 (when water loss was negligible, at 12.5% FC).

#### Flower and pod tagging

The start of flowering and podding was recorded for plants in each container, and the time to 50% flowering and 50% podding was recorded when there were ≥3 plants per container with at least one flower or one pod. All new flowers and pods from one plant in each container (i.e. two plants per genotype per treatment in each replicate not used for destructive measurements) were tagged every 2 d, with the date of flowering and podding noted on the tags.

#### Leaf water potential and gas exchange

The pre-dawn leaf water potential of young fully expanded leaves on primary branches was measured (04:00–05:00h WST) in a pressure chamber (Soil Moisture Equipment Corp., Santa Barbara, CA, USA). On similar leaves to those used for leaf water potential, measurements of gas exchange were carried out between 10:30h and 12:00h WST using a LICOR-6400 with a red/blue LED light source (LI-COR, Lincoln, NE, USA). Photosynthetic photon flux density at the leaf surface was set at 1500 μmol m^−2^ s^−1^, block temperature at 25 °C, flow rate at 500 μmol s^−1^, and ambient CO_2_ concentration of the incoming gas stream at 380 μmol mol^−1^ as in Pang *et al.* (2011).

#### In vitro *pollen viability and germination*

Pollen viability, germination and pollen tube growth were determined at 0, 3, 9, and 15 d after the water treatments were imposed when the soil water content was 80% (1.0 FTSW), 69% (0.83 FTSW), 46% (0.50 FTSW), and 25% FC (0.18 FTSW), respectively, in the WS containers. For pollen viability, pollen from five hooded ﬂowers for each genotype, treatment, and replicate was collected and pooled in 2ml Eppendorf tubes by squeezing the keel from the base upwards with forceps until most pollen exuded through the tip. A 1ml aliquot of 10% sucrose was immediately added to the freshly collected pollen. Pollen viability was examined within 2h after sampling using the fluorochromatic reaction according to Heslop-Harrison and Heslop-Harrison (1970). Pollen viability was assessed under a ﬂuorescence microscope (Carl Zeiss Pty Ltd, Oberkochen, Germany); pollen grains with grey colour, rather than bright blue, were considered non-viable. The percentage of viable and non-viable pollen was measured by examining 300 pollen grains per sample.

For the determination of *in vitro* pollen germination, pollen collected from another five similar flowers was mixed with 2ml of pollen culture medium [10% sucrose, 100 μg g^–1^ H_3_BO_3_, 300 μg g^−1^ Ca(NO_3_)_2_·4H_2_O, 200 μg g^−1^ MgSO_4_·7H_2_O, and 100 μg g^−1^ KNO_3_] and incubated at 25 °C in the dark for 4h (Brewbaker and Kwack, 1963). The process was halted by adding one drop of acetic alcohol (glacial acetic acid:ethanol, 1:3, v/v) to the samples as a fixative. Pollen was recorded as germinated when the length of the pollen tube exceeded the diameter of the pollen grain. The percentage of pollen germination was estimated by examining 300 pollen grains per sample.

#### In vivo *pollen tube growth*

The 10 flowers used for the *in vitro* pollen viability and germination were collected to observe *in vivo* pollen tube growth at 25 °C according to Mori *et al.* (2006). The flowers with petals removed were fixed in acetic alcohol (glacial acetic acid:ethanol, 1:3 v/v) for 24h, followed by fixing in 70%, then 50%, and then 30% ethanol for 0.5h for each step, and washed with distilled water for another 0.5h. The samples were then cleared with 8M NaOH overnight, and thoroughly rinsed with distilled water before being stained with decoloured aniline blue solution for 24h in the dark. Using a ﬂuorescence microscope and UV irradiation (Carl Zeiss Pty Ltd), the total number of pollen tubes in each pistil was counted and the observation of whether a pollen tube had reached the ovary was noted.

#### Determination of embryo size

Nine days after the two water treatments were imposed when soil water content in the WS treatment was 46% FC (0.50 FTSW), flowers that opened on this day were tagged in both the WW and WS treatments. Seven days later (16 d after the imposition of the two treatments), when soil water content in the WS treatment was 22% FC (0.14 FTSW), 10 young pods (~3mm long) developed from the tagged flowers were sampled to measure the embryo size in the WW and WS treatments. The WS plants had both yellow and green pods. The number of yellow and green pods was counted. The embryo was dissected from the young pods with a scalpel under an anatomical dissecting microscope (Carl Zeiss Pty Ltd) and photographed with a Zeiss AxioCam HRm microscope camera. The projected area of the embryo was determined with Image J software (US National Institutes of Health, Bethesda, MD, USA).

#### Seed growth rate

Seed growth rate was determined for pods which developed from flowers tagged at 98 DAS (2 d prior to the imposition of water treatments) when the soil water content was at 80% FC (1.0 FTSW). Five pods developed from the flowers tagged at 98 DAS were randomly selected and sampled at 9, 16, 23, 30, and 37 days after flowering (DAF), and at physiological maturity (50 DAF for the WS treatment and 64 DAF for the WW treatment) in each genotype, treatment, and replicate. Pods were separated into pod wall and seeds, except that pods at 9 DAF were too small to separate. The number of seeds from five pods was counted. The dry weight of the pod wall and seed was recorded after samples were oven-dried at 60 °C for 72h.

Seed dry weight data were fitted by non-linear regression to a logistic curve (Darroch and Baker, 1990; Davies *et al.*, 1999):

$$\text{Seed dry weight}=A/\left[\text{1}+{\text{exp}}^{\left(B-Ct\right)}\right]$$

where *A* estimates the final seed weight, *B* is related to both the duration and rate of seed growth, *C* is related to the rate of seed growth, and *t* is time in days. Seed growth rate was calculated using the following equation (Darroch and Baker, 1990):

dy/dx=C×y×(A−y)/A

where d*y*/d*x* represents the instantaneous rate of pod wall and seed growth. This value reaches a maximum when *y*=0.5×*A*; thus, the maximum rate of seed growth (*R*) was calculated as: *R*=*C*×*A*/4 (Darroch and Baker, 1990; Davies *et al.*, 1999). Duration of seed growth was defined as the time required for each seed to reach 95% of its final dry weight and calculated as: *T*=(*B*+2.944)/*C* (Darroch and Baker, 1990; Davies *et al.*, 1999).

#### ABA quantification

For the determination of ABA concentration in pod and seed tissue, flowers were tagged when the soil water content was at 80% (2 d before the imposition of the water treatments, 1.0 FTSW) and 58% FC (6 d after the water treatments were imposed, 0.66 FTSW), and pods developed thereafter were sampled at 9, 16, and 23 DAF. Each time, five pods were sampled for each genotype and each treatment per replicate. Since the pods that developed from flowers that were tagged at 58% FC senesced after 16 DAF, no samples were taken thereafter. Pods were separated into pod wall and seed, except for those pods at 9 DAF which were too small to separate. The samples were immediately wrapped in foil, snap-frozen in liquid N_2_, and stored in a freezer at –80 °C. The samples were later ground to a fine powder under liquid N_2_, and 50–100mg of frozen tissue was weighed into chilled 2ml Eppendorf tubes. The extraction of ABA followed the protocol of Dave *et al.* (2011). The extraction was analysed on an optimized Agilent 6530 Accurate Mass Q-TOF LC/MS (Agilent Technologies Inc., Santa Clara, CA, USA). Details of the operating parameters of LC/MS were described in Miyazaki *et al.* (2014). Data were acquired for three subsamples for each sample and analysed using Agilent Technologies MassHunter Qualitative Analysis software (version B.05.00).

#### Harvest procedure

At physiological maturity (150 and 164 DAS for the WS and WW plants, respectively), the one plant per container that was not used for destructive sampling was cut at ground level and partitioned into leaves, stems, and pods. Leaves and stems were oven-dried at 60 °C for 48h and weighed. All pods with the same podding date were combined, separated into pod wall and seeds for counting, and weighed after drying at 30 °C for 7 d. The number of flowers and total pods was calculated from tags with flowering and podding dates, respectively. Flower abortion was calculated from tags where no podding date was recorded. The percentage of flower abortion was calculated as the number of aborted flowers (tagged, but with no podding date) relative to the total flowers (total number of tags). Abscised pods were calculated from tags where podding date was recorded, but no pods were present at maturity. The percentage of abscised pods was calculated as the number of abscised pods relative to the total number of pods as recorded on the tags. Empty pods were calculated from tags where pods were present but had <40% of the average size (Leport *et al.*, 2006) or no seed present at maturity. The percentage of empty pods was calculated as the number of empty pods relative to the total number of pods as recorded on the tags. Harvest index was calculated as the ratio of seed weight to above-ground dry weight.

### Statistical analysis

For the field yield data, linear mixed models have been formulated based on a randomization model approach (Smith *et al.*, 2005) and ASReml-R (Butler *et al.*, 2009) was used for the analysis. The model for the response variable yield includes blocking terms to account for the randomization process and additional terms to model the extra sources of variation, such as spatial trends and extraneous variation. The seed yield of each genotype at each site was predicted using the best linear unbiased predictions (BLUPs) accounting for the spatial variation and covariates where appropriate. The statistical analysis for yield aimed at modelling the genotype by environment (G×E) interaction and Smith *et al.* (2005) approach for the analysis of multienvironment trials (METs) was adopted. We fitted the environment effect as fixed and the G×E effect as random. The data were analysed using ASReml-R (Butler *et al.*, 2009) which facilitates joint modelling of blocking structure, spatial and extraneous variation, treatment effects, and modelling the covariance structure of GxE effect.

For the glasshouse experiment, a two-factorial (genotype and water treatment) randomized complete block design was used where the blocking structure consisted of three replicates. Data for growth and other parameters were analysed using general ANOVA in Genstat version 15.2 (Lawes Agricultural Trust, Rothamsted Experimental Station, UK, 2012). The model also accounted for the blocking structure. The statistical model with FTSW as the explanatory variable used a split-line regression in Genstat to assess the FTSW threshold values for cumulative flower number, cumulative total pod number, cumulative filled pod number, cumulative seed number, pre-dawn leaf water potential, leaf photosynthetic rate, stomatal conductance, and leaf transpiration rate. The seed dry weight accumulation data were modelled by fitting non-linear regression using SigmaPlot version 13.0 (2014 Systat Software, Inc.).

## Results

### Field experiment

Seed yield varied greatly among genotypes at all three sites, ranging from 560kg ha^−1^ to 1200kg ha^−1^ at York in 2012, from 500kg ha^−1^ to 1060kg ha^−1^ at Bindi Bindi in 2013, and from 890kg ha^−1^ to 1780kg ha^−1^ at Cunderdin in 2013 (see Supplementary Fig. S2). The genotypic effect was significant (*P*<0.001) at all sites. There was a significant G×E interaction in seed yield across the three sites over 2 years (*P*<0.001). Mean seed yield at Cunderdin was ~55% and 76% higher than that at York and Bindi Bindi, respectively (Supplementary Fig. S2). In both years, DICC8172 had consistently higher yields than DICC8156, being 19% (*P*>0.05, not significant), 40% (*P*<0.05), and 24% (*P*<0.05) higher at York, Bindi Bindi, and Cunderdin, respectively. These two genotypes with similar phenology (50% flowering in DICC8172 was ~4–5 d earlier than that of DICC8156), were selected for the detailed glasshouse study.

### Glasshouse experiment

#### Phenology

Flowering commenced at 67 and 78 DAS, 50% flowering was at 73 and 83 DAS, podding commenced at 88 and 92 DAS, and reached 50% podding at 93 and 94 DAS in DICC8172 and DICC8156, respectively. The first flowers in both genotypes and both water treatments failed to produce a pod. The time from 50% flowering to 50% podding was 20 d and 11 d for DICC8172 and DICC8156, respectively. The water treatment was imposed at 100 DAS, 8–12 d after podding commenced. No difference in flowering time or podding time was found between plants assigned to the WW and WS treatments. The WS plants of both DICC8172 and DICC8156 reached physiological maturity at 144 DAS, 9 d earlier than those of the WW plants (163 DAS).

#### Change in soil water content

**Fig. 1.**
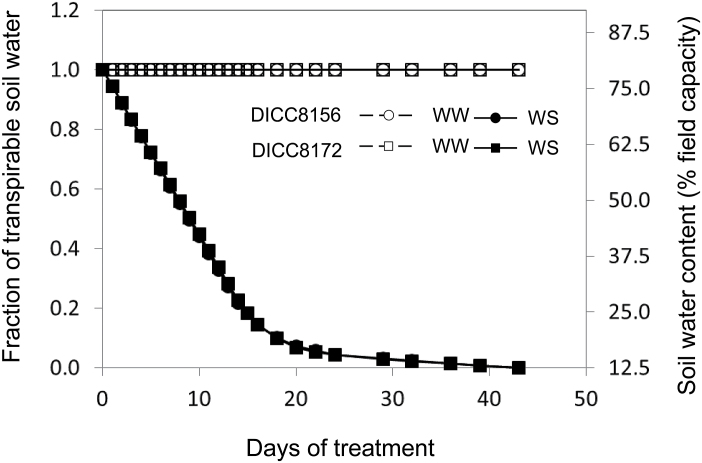
The soil water content, as a percentage of field capacity and fraction of transpirable soil water in the well-watered (WW) and water-stressed (WS) treatments with time after the start of the water treatments (100 DAS) in two chickpea genotypes, DICC8156 and DICC8172. Data are means ±SE (n=3). There was a significant effect of water treatment (*P*<0.001). Data from two containers per replicate were pooled and the mean per container used. Note that SE values are less than the size of the symbol in all cases.

The FTSW in the WW treatment was maintained at 1.0 by watering the containers daily to 80% of FC (Fig. 1). As the loss for each container was restricted to a maximum of 750ml d^–1^, the soil water content (FTSW) in the WS treatment decreased steadily for the first 15 d after water was withheld (Fig. 1). After 15 d of treatment, when the water loss per container was <750ml per day and no water was added back, FTSW decreased slowly, to reach ~0 (12.5% FC) after 36 d of treatment. During the WS treatment, no genotypic differences in the dynamics of FTSW were observed (Fig. 1).

No difference in volumetric soil water content with soil depth was observed between the two genotypes (data not shown); therefore, the combined volumetric soil water content was used for further analysis. The volumetric soil water content at different soil depths varied in the two water treatments, with lower values in the upper soil layers and higher values at increasing depth, even in the WW treatment maintained at 80% FC (Supplementary Fig. S3). The volumetric soil water content in the WW treatment was maintained at 0.16 m m^−3^ in the upper 0–0.1 m and increased to ~0.32 m m^−3^ in the 0.5–0.6 m depth. In the WS treatment, the volumetric soil water content at all soil depths decreased continuously over the first 16 d, suggesting that roots actively withdrew water throughout the soil profile, followed by little change thereafter, with values of 0.04 m m^−3^ in the upper 0–0.1 m and 0.08–0.11 m m^−3^ at other depths at the end of the experiment (Supplementary Fig. S3).

#### Pre-dawn leaf water potential, leaf photosynthetic characteristics, and their FTSW breakpoint values

**Fig. 2.**
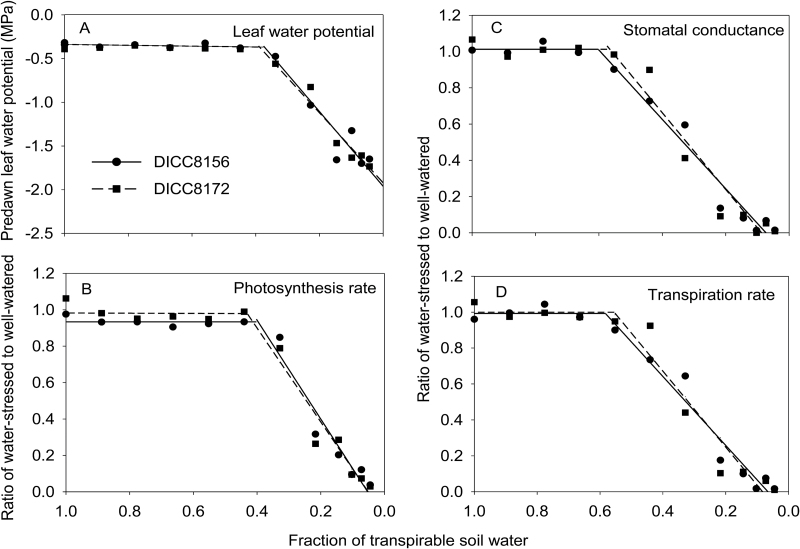
Split-line regression between the fraction of transpirable soil water content in the water-stressed plants of two chickpea cultivars, DICC8156 and DICC8172, and pre-dawn leaf water potential (A), rate of leaf photosynthesis (B), stomatal conductance (C), and rate of leaf transpiration (D), showing break point values where the slope of the fitted regression changed significantly. Data in B, C, and D are relative to the well-watered control and all are means of three replicates.

The pre-dawn leaf water potential in the WW plants was maintained at –0.3MPa to –0.4MPa (Supplementary Fig. S4A). In the WS treatment, the pre-dawn leaf water potential was maintained similar to that in the WW treatment for the first 10 d after the treatments were imposed when FTSW was >0.40 (Fig. 2A; Supplementary Fig. S4). When the FTSW fell below 0.37 and 0.39 for DICC8172 and DICC8156, respectively, the pre-dawn leaf water potential decreased for the next 6 d (Fig. 2A; Table 2) to stabilize at about –1.7MPa when FTSW fell below 0.10 in both genotypes.

The rates of leaf net photosynthesis in both the WW and WS plants were ~30 µmol m^−2^ s^−1^ in DICC8156 and ~32 µmol m^−2^ s^−1^ in DICC8172 for the first 10 d after the water treatments were imposed (Supplementary Fig. S4B). In the WS treatment, the rates of leaf net photosynthesis then decreased when FTSW fell below 0.40 in DICC8156 and 0.43 in DICC8172 (Fig. 2B; Table 2). The photosynthetic rates reached close to zero after 24 d of treatment when FTSW decreased to 0.04 (Fig. 2B; Supplementary Fig. S2B). The stomatal conductance was 0.20–0.25mol m^−2^ s^−1^ and leaf transpiration was 5–6 mmol m^−2^ s^−1^ in the WW plants, and remained at these values in the WS plants for the first 10 d after water was withheld, when both the stomatal conductance and leaf transpiration rate began to decrease (Supplementary Fig. S4C, D) when the FTSW values fell to ~0.58 for both genotypes (Figs. 2C, D; Table 2). The WW plants of DICC8172 had a higher photosynthetic rate (*P*<0.001), stomatal conductance (*P*<0.01), and transpiration rate (*P*<0.01) than the WW plants of DICC8156 after 112 DAS (12 d after the water treatments were imposed, Supplementary Fig. S4).

#### Flower production and abortion

**Fig. 3.**
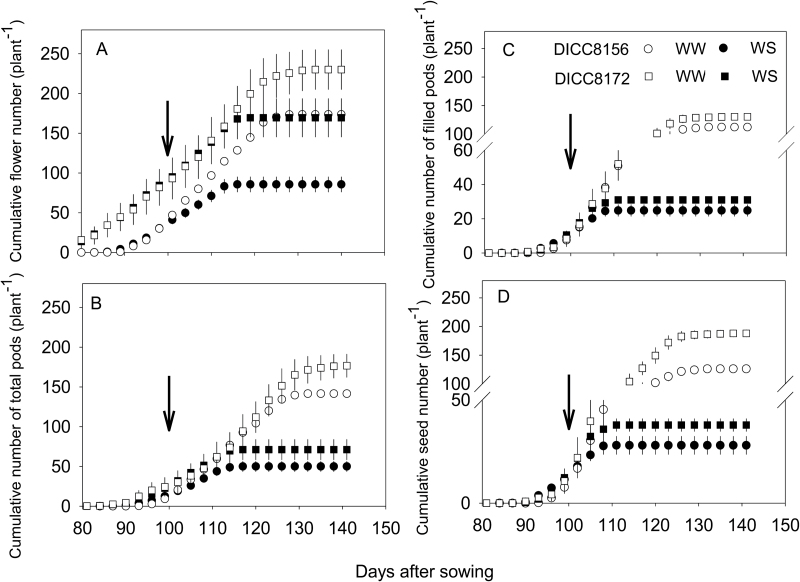
Changes in the cumulative number of flowers (A), total pods (B), filled pods (C), and seeds (D) per plant with time (days) after sowing in well-watered (WW) and water-stressed (WS) treatments and two chickpea genotypes, DICC8156 and DICC8172. Date are means ±SE (*n*=3) from two plants pooled per replicate. Arrows indicate the start of water treatments.

At the start of the water treatments (100 DAS), plants of DICC8172 had developed more flowers (90 per plant) than DICC8156 (40 per plant) (Fig. 3A). Flower production in both genotypes was reduced by the WS treatment (*P*<0.01, Fig. 3A; Table 1). In the WW DICC8172, plants produced a total of 230 flowers per plant, and flower production ceased 31 d after the water treatments were imposed (131 DAS) (Fig. 3A; Table 1) even though water was still available (Supplementary Fig. S3). In the WS treatment, DICC8172 plants continued to produce flowers at a similar rate to that in WW plants for 18 d after the beginning of the WS treatment (118 DAS), when flowering stopped after reaching 170 flowers per plant. For DICC8156, the rate of flower production in the WS treatment was lower than in the WW treatment immediately after the treatments were imposed (Fig. 3A). In the WS plants, flowering in DICC8156 stopped 16 d after the treatments were imposed (116 DAS) when the chickpeas had 86 flowers per plant, and stopped in the WW plants 31 d after treatments were imposed (131 DAS) when the chickpeas had 174 flowers per plant (Fig. 3A). Thus, water stress reduced flower numbers by half and by a quarter of those in the WW controls in DICC8156 and DICC8172, respectively (*P*<0.01). In the WS treatment, the cumulative flower number in both genotypes increased linearly, until FTSW decreased to 0.21 for DICC8156 and 0.13 for DICC8172 (Supplementary Fig. S5; Table 2).

**Table 1.** Above-ground dry weight, seed yield, harvest index, yield components, percentage flower abortion, percentage abscised pods, and percentage empty pods in well-watered and water-stressed genotypes of chickpea, DICC8156 and DICC8172, at physiological maturity

	Well-watered		Water-stressed		Genotype	Water	Genotype ×water
	DICC8156	DICC8172	DICC8156	DICC8172			
Above-ground dry weight (g per plant)	87.8±5.8	93.7±3.7	40.1±6.0	41.3±2.0	NS	***	NS
Seed yield (g per plant)	31.4±1.1	35.6±1.1	4.6±0.5	5.5±0.2	*	***	NS
Harvest index	0.36±0.02	0.38±0.00	0.12±0.02	0.13±0.00	NS	***	NS
Number of flowers (per plant)	174±7	230±25	86±10	170±24	**	**	NS
Number of aborted flowers (per plant)	31±4	54±12	36±5	98±17	**	*	NS
Percentage of flower abortion	18±2	23±3	42±4	58±5	*	***	NS
Number of total pods (per plant)	142±3	177±15	50±7	71±13	**	***	NS
Number of abscised pods (per plant)	17±3	18±2	11±0	15±3	NS	NS	NS
Percentage of abscised pods	12±2	10±0	23±3	21±2	NS	**	NS
Number of filled pods (per plant)	112±2	130±5	25±3	31±1	**	***	NS
Number of empty pods (per plant)	13±2	28±8	14±4	25±9	*	NS	NS
Percentage of empty pods	9±1	15±3	27±4	32±8	NS	**	NS
Number of seeds (per plant)	126±5	188±6	28±4	38±3	***	***	**
Mean seed weight (mg per seed)	266±14	189±1	168±7	145±7	**	***	**

Data are means ±SE (*n*=3).

*P<0.05; **P<0.01; ***P<0.001; NS, no significant difference

**Table 2.** Values of the fraction of transpirable soil water content at which the listed numbers and processes began to decrease in the water-stressed treatment in two chickpea genotypes, DICC8156 and DICC8172

	DICC8156	DICC8172	Significance
Cumulative flower number	0.21±0.03	0.13±0.03	NS
Cumulative total pod number	0.15±0.02	0.15±0.03	NS
Cumulative filled pod number	0.57±0.06	0.57±0.04	NS
Cumulative seed number	0.57±0.06	0.57±0.05	NS
Pre-dawn leaf water potential	0.37±0.03	0.39±0.03	NS
Photosynthetic rate	0.40±0.04	0.43±0.02	NS
Stomatal conductance	0.60±0.03	0.58±0.04	NS
Transpiration rate	0.58±0.02	0.57±0.02	NS

Data are means ±SE (*n*=3).

NS, no significant difference.

While the number of aborted flowers of DICC8156 was similar in the WS treatment to that in the WW treatment, the absolute number of aborted flowers in WS DICC8172 increased by 44 per plant compared with the WW plants (Table 1). The percentage flower abortion was 2.3- and 2.5-fold higher in the WS plants than in the WW plants of DICC8156 and DICC8172, respectively (Table 1).

Significant effects of genotype were found for flower number (*P*<0.01), aborted flower number (*P<*0.01), and percentage of flower abortion (*P*<0.05), but no significant genotype×water treatment two-way interactions were observed (*P* > 0.05) (Table 1). DICC8172 had 54% more flowers, 123% more aborted flowers, and a 33% greater percentage of flower abortion than DICC8156 (Table 1).

#### Pod and seed production, abscised pods, and empty pods

In the WW plants, more flowers in DICC8172 resulted in more total pods and filled pods than in DICC8156 (Table 1). Water stress reduced pod and seed production (*P*<0.0001, Fig. 3B–D; Table 1). At the start of the water treatments, DICC8172 had more total pods (26 per plant) than DICC8156 (15 per plant) (Fig. 3B). For the first 16 d and 17 d of treatment, the rate of pod production was higher in DICC8172 (2.8 pods d^−1^) than in DICC8156 (2.3 pods d^−1^), after which no more pods were produced in the WS treatment, giving a total of 50 pods per plant in DICC8156 and 71 pods per plant in DICC8172, that is only 35% of the 142 pods per plant and 40% of the 177 pods per plant in the WW plants of the two genotypes (*P<*0.001), respectively. However, the number of abscised pods was similar in both WW and WS treatments for both genotypes (*P*>0.05), resulting in the percentage of abscised pods in the WS treatment (22%) being about twice that in the WW treatment (11%) for both genotypes (*P*<0.01).

At the start of water treatments, DICC8156 and DICC8172 had similar numbers of filled pods (11 per plant) and seeds (13 per plant for DICC8156 and 14 per plant for DICC8172) (Fig 3C, D). The number of filled pods in DICC8156 and DICC8172 stopped increasing 9 d after the initiation of the water treatments (109 DAS) (Fig. 3C) when FTSW decreased to 0.57 (Table 2; Supplementary Fig. S5). The faster rate of pod production in the WS treatment in DICC8172 than in DICC8156 resulted in 31 and 25 filled pods per plant, respectively, compared with 130 and 112 filled pods per plant in the WW plants (Fig. 3C; Table 1). No effect of water treatment on the number of empty pods was found in either genotype, thus the percentage of empty pods to total pods in the WS treatment was 3- and 2.1-fold higher than in the WW treatment for DICC8156 and DICC8172, respectively (*P*<0.01). Similarly, the number of seeds per plant in the WS treatment decreased significantly to 28 and 38 per plant (22% and 20% of the WW controls) in DICC8156 and DICC8172, respectively, compared with 188 and 126 per plant in the WW plants (Fig. 3D; Table 1). There was a significant genotype×water treatment interaction for seed number per plant (*P*<0.01) (Table 1). In the WW treatment, mean seed number per filled pod was higher at 1.45 (45% of pods had two seeds) in DICC8172 than in DICC8156 (1.12 seeds per pod), whereas in the WS treatment seed number per pod decreased to 1.22 in DICC8172, but was unchanged at 1.12 seeds per pod in DICC8156.

Significant effects of genotype were found on the numbers of total pods, filled pods, and empty pods (Table 1). While DICC8172 had a 29% higher number of total pods, 17% more filled pods, and 86% more empty pods than DICC8156, no significant genotype×water treatment interaction was found in any of these parameters (*P*>0.05) (Table 1).

In the WS treatment, FTSW decreased to 0.15 before the number of total pods stopped increasing, but decreased to only 0.57 before seed set halted, and seed numbers and filled pod numbers did not increase in either genotype (Supplementary Fig. S5; Table 2).

#### Above-ground dry weight, seed yield, harvest index, and seed size

At maturity, total above-ground dry weight decreased significantly from ~90g per plant in the WW plants to ~40g per plant in the WS plants (*P*<0.001), while seed yield was reduced by ~85% in both genotypes (*P*<0.001) (Table 1). Taking an average of the WW and WS treatments, DICC8172 had a 14% greater (*P*<0.05) seed yield than DICC8156 (Table 1). Harvest index decreased from ~0.37 in the WW treatment to ~0.13 in the WS treatment in both genotypes (*P*<0.001). The genotype×water treatment interaction for total above-ground dry weight and harvest index was not significant (*P* > 0.05) (Table 1).

**Fig. 4.**
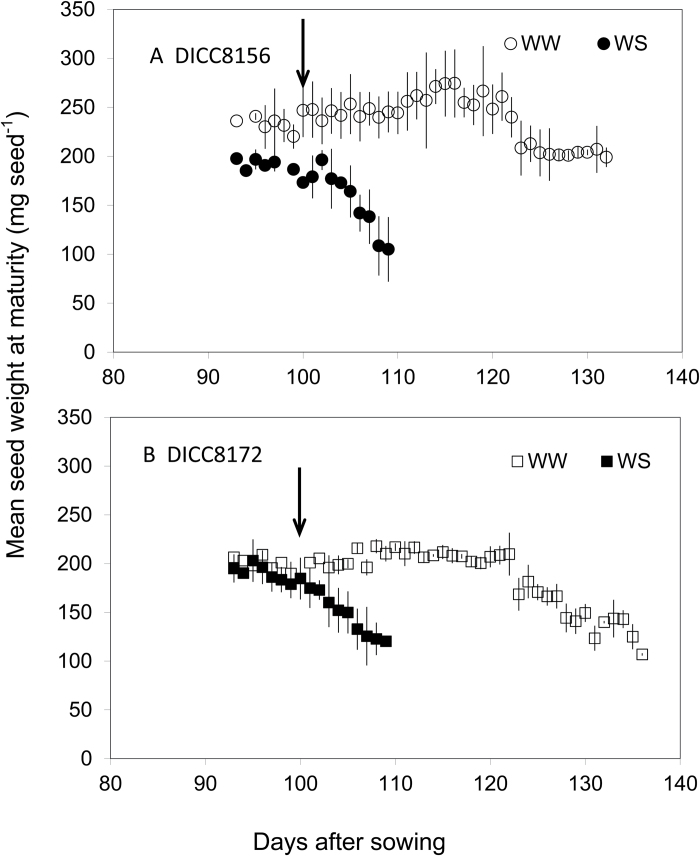
Change in mean seed weight (seed size) at maturity in pods set at different times (days) after sowing in well-watered (WW) and water-stressed (WS) treatments and two chickpea genotypes, DICC8156 (A) and DICC8172 (B). Data are means ±SE (*n*=3) from two plants pooled per replicate. Arrows indicate the start of the water treatments.

At physiological maturity, seed size (mean seed weight, Table 1) in WW plants of DICC8156 and DICC8172 was 266mg and 189mg per seed, respectively, while the corresponding values in the WS plants were 168mg (63% of the WW control) and 145mg per seed (77% of WW control), resulting in a significantly (*P*<0.001) greater reduction in DICC8156 than in DICC8172 (Table 1). These mean values of seed size hide considerable variation among seeds from different times of podding during the WS treatment. In DICC8172, the cohort of pods initiated between 93 and 100 DAS (i.e. before the water treatments were imposed), had similar seed sizes at maturity in both the WS and WW plants, presumably as much of this seed growth would have occurred during the slow decrease in soil water content and before the leaf water potential and rate of photosynthesis began to decrease in the WS treatment (Fig. 4). However, in DICC8156, all pods initiated between 93 and 102 DAS (before water was withheld, but subject to increasing water stress during seed growth) had ~20% smaller seeds than those in WW plants (Fig. 4). In both genotypes, seed size at maturity in all pods initiated after 101 DAS decreased with podding date in the WS plants, while there was no decrease until late in plant development in the WW treatment (Fig. 4). In the cohort of pods initiated at 109 DAS (the last pods to develop and produce a seed in the WS plants), seed size in the WS plants was reduced to 42% (DICC8156) and 57% (DICC8172) of that in WW plants. Due to the withholding of water after 144 DAS in the WW plants, seed size in DICC8172 at maturity in pods initiated between 122 and 136 DAS decreased with podding date; seed size in DICC8156 in pods initiated between 123 and 132 DAS also decreased.

#### Pod wall and seed growth rates

**Fig. 5.**
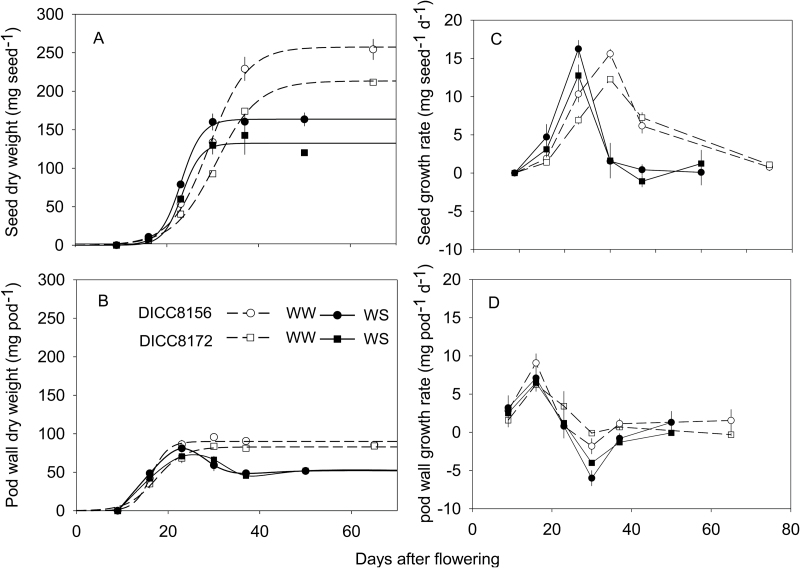
Changes with time (days after flowering) in the dry weight of seeds (A) and pod walls (B), and the growth rate of seeds (C) and pod walls (D) in two chickpea genotypes, DICC8156 and DICC8172, in both well-watered (WW) and water-stressed (WS) treatments. Pods were developed from flowers tagged 2 d prior to the imposition of the water treatments. Data are means ±SE (*n*=3) of five pods pooled per replicate. Logistic curves were fitted to seed dry weight data. There was a significant effect of water treatment on final seed weight (*P*<0.001, LSD_0.05_=24mg per seed); while there was a significant effect of genotype for final seed weight (*P<*0.01, LSD_0.05_=24mg per seed) and maximum seed growth rate (*P* <0.05, LSD_0.05_=3mg d^−1^).

For pods developed from flowers which were tagged 2 d before water stress was imposed when FTSW was 1.0, the pod wall grew more rapidly than the seed (Fig. 5). The maximum rate of pod wall growth occurred at 16 DAF, with the pod wall reaching its maximum dry weight at 23 DAF in both WS and WW plants in both genotypes (Fig. 5B, D). After 23 DAF, the pod wall dry weight changed little in the WW chickpea (Fig. 5B), but decreased by 40% and 36% in DICC8156 and DICC8172, respectively, in the WS plants (Fig. 5B). Seed dry weight followed a sigmoid growth curve (Fig. 5A). There was a lag phase with little increase in seed dry weight for the first 16 d after flowering, then a period of rapid seed growth followed by a slow increase when seed growth was close to completion (Fig. 5A). Water stress induced earlier seed growth, with the maximum rate at 23 DAF in the WS treatment and at 30 DAF in the WW treatment (Fig. 5C). While DICC8156 had a higher maximum rate of seed growth at 16mg d^−1^ than DICC8172 at 12mg d^−1^ (*P*<0.05), the maximum rate of seed growth was similar in the WW and WS treatments (Fig. 5C). The duration of seed growth in the WS treatment was ~29 d in both genotypes, which was ~30% (*P<*0.01) less than the duration in the WW plants. The final seed size estimated from the logistic curve (Fig. 5A) was similar to the final mean seed weight of seeds developed through the entire experimental period (Table 1).

#### Pollen viability, pollen germination, pollen tube growth, and embryo size

**Fig. 6.**
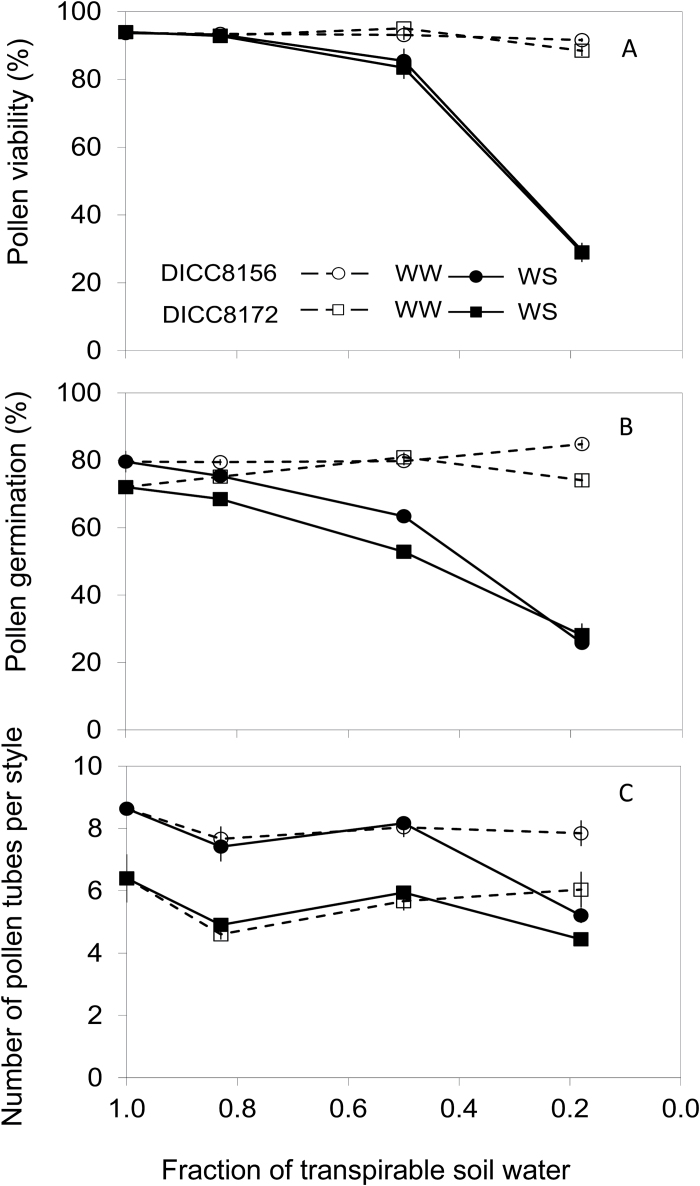
Changes in percentage pollen viability (A), percentage *in vitro* pollen germination (B), and number of pollen tubes per style (C) with the fraction of transpirable soil water (FTSW) in the water-stressed (WS) treatment of two chickpea genotypes, DICC8156 and DICC8172. FTSW was maintained at 1.0 in the well-watered (WW) plants, but the pollen characteristics were measured on the same days as the WS plants. For pollen viability, there was a two-way interaction between water treatment and FTSW (*P<*0.001, LSD_0.05_=4%). For pollen germination, there was a three-way interaction of genotype×water treatment×FTSW (*P*<0.001, LSD_0.05_=5%). For the number of pollen tubes per style, there was a significant effect of genotype (*P*<0.001, LSD_0.05_=0.4) and a two-way interaction between water treatment and FTSW (*P*<0.001, LSD_0.05=_0.8). No other effects or interactions were significant. Date are means ±SE (*n*=3). Pollen from five flowers per replicate was pooled for pollen viability and pollen germination, while 10 flowers per replicate were pooled for the number of pollen tubes per style.

In the WW treatment, pollen viability was ~90% in both genotypes and did not vary significantly over the sampling period (Fig. 6). In the WS treatment, pollen viability decreased to ~84% at 0.50 FTSW (9 d after withholding water), and to ~30% at 0.18 FTSW (15 d after withholding water) in both genotypes (Fig. 6A). Measured pollen viability did not guarantee pollen germination. In the WW treatment, 80–85% of the pollen grains germinated after 4h incubation in DICC8156 and 72–81% in DICC8172, at all sampling times (Fig. 6B), while, in the WS treatment, 63% germinated for DICC8156 and 53% for DICC8172 in flowers sampled at 0.50 FTSW (9 d after withholding water), and ~27% germinated in both DICC8156 and DICC8172 for flowers sampled at 0.18 FTSW (Fig. 6B).

In the WW treatment, DICC8156 had ~8 germinated pollen tubes per pistil *in vivo* compared with ~6 germinated pollen tubes per pistil in DICC8172 throughout the sampling period (Fig. 6C). Water stress only significantly decreased the *in vivo* pollen tube number when the soil water content decreased to 0.18 FTSW (15 d after withholding water), when the number of germinated pollen tubes was 5.2 in DICC8156 and 4.4 in DICC8172. Averaging the two water treatments, DICC8156 had more *in vivo* pollen tubes per pistil than DICC8172 (*P<*0.001). Although pollen viability and germination decreased significantly at 0.18 FTSW, at least one pollen tube was observed reaching the ovary in both genotypes (Supplementary Fig. S6). Moreover, the embryo size of green pods in the WS treatment at 7 DAF developed from flowers tagged when the FTSW was 0.50 (the FTSW at the time of sampling was 0.14) was similar to that in WW plants (0.22mm^2^ projected area in DICC8156 and 0.32mm^2^ projected area in DICC8172) at the same age; however, 16 d after flowering the pods in the WS treatment were already small and senescing (Supplementary Fig. S7) and at maturity produced no pods or seeds. Moreover, in the WS treatment, 56% and 23% of the pods in DICC8156 and DICC8172, respectively, were yellow, not green, and the embryo size in these pods was smaller (0.09mm^2^ in DICC8156 and 0.13mm^2^ projected area in DICC8172).

#### Abscisic acid

The ABA concentration in young pods at 9 DAF developed from flowers tagged 2 d before the water stress was imposed when FTSW was 1.0, and measured 9 d later when the FTSW in the WS treatment was 0.61, increased by ~20% compared with that in WW plants; and the ABA concentration in DICC8156 was almost twice that in DICC8172 in both WW and WS plants (Table 3). The ABA concentration of pods at 9 DAF, developed from flowers tagged at 0.66 FTSW and measured in WS plants when the FTSW was 0.18, increased significantly, being ~6-fold higher than that in WW plants, and ~4.5-fold higher than the pods sampled when the FTSW was 0.61 in both genotypes (Table 3).

**Table 3.** Concentration of abscisic acid in entire young pods at 9 days after flowering (DAF), and the pod wall and seed of pods at 16 and 23 DAF Pods were developed from flowers tagged in both well-watered (WW) and water-stressed (WS) plants when the fraction of transpirable soil water (FTSW) of the WS plants was either 1.0 or 0.66 for pods at 9 DAF, and was 1.0 for pods at 16 and 23 DAF. FTSW was maintained at 1.0 in the WW treatment. The FTSW at the time of sampling is given. There was a three-way interaction of genotype×water treatment×FTSW (*P*<0.05, LSD_0.05_=693ng g^−1^) in the pods at 9 DAF, and of genotype×water treatment×DAF for seed (*P*<0.05, LSD_0.05_=460ng g^−1^). For pod wall, two-way interactions of genotype×water treatment (*P*<0.05, LSD_0.05_=163ng g^−1^), genotype×DAF (*P*<0.01, LSD_0.05_=163ng g^−1^), and water treatment×DAF (*P*<0.01, LSD_0.05_=163ng g^−1^) were significant, but the three-way interaction was not found

Days after flowering	Abscisic acid concentration (ng g^−1^ FW)					
	Well-watered			Water-stressed		
	FTSW when flowers were produced and pods were sampled	DICC8156	DICC8172	FTSW when flowers were produced and pods were sampled	DICC8156	DICC8172
Pod wall+seed						
9	1.00, 1.00	907±166 a	474±32 a	1.00, 0.61	1157±225 a	620±103 a
9	1.00, 1.00	781±97 a	622±57 a	0.66, 0.18	5197±425 c	2907±524 b
Pod wall						
16	1.00, 1.00	544±22	345±49	1.00, 0.18	1493±155	840±49
23	1.00, 1.00	76±18	74±4	1.00, 0.05	405±97	322±44
Seed						
16	1.00, 1.00	625±44 a	637±87 a	1.00, 0.18	1746±41c	1451±297 bc
23	1.00, 1.00	1236±68 b	1260±178 b	1.00, 0.05	3008±461d	3969±69 e

Data are means ±SE (*n*=3). Five pods per replicate were pooled

In the WW plants, the ABA concentration in pod wall and in seeds at 16 and 23 DAF was similar between the two genotypes, with the ABA concentration decreasing in the pod wall and increasing in the seed over the 7 d between sampling (Table 3). In both genotypes, the ABA concentration in the pod wall and seed, developed from flowers tagged at 1.0 FTSW, increased significantly in response to water stress at 16 and 23 DAF, being 2.7- to 5.3-fold higher than in WW plants (Table 3). When the rate of growth of the pod wall began to decrease (Fig.5D) between 16 and 23 DAF, the ABA concentration in the pod wall decreased significantly, despite the FTSW decreasing from 0.18 to 0.05; whereas the ABA concentration in the seed increased significantly (Table 3), coinciding with the maximum growth rate of the seed in the WS plants (Fig. 5C).

## Discussion

Terminal drought, imposed as controlled progressive soil drying from early podding on two genotypes of chickpea exhibiting differences in yield under dryland conditions in the field, significantly reduced above-ground biomass, reproductive growth, harvest index, and seed yield. The terminal drought at least doubled the percentage of flower abortion, pod abscission, and number of empty pods. While flowers and pods continued to be produced until low soil water contents of ~0.2 FTSW remaining, pods containing a seed ceased to be produced at ~0.6 FTSW remaining, a similar FTSW threshold to the value at which stomatal conductance and transpiration began to decrease and ABA reached high concentrations in the developing pods. The pre-dawn leaf water potential and net photosynthesis began to decrease at ~0.4 FTSW remaining, while the pods still grew slowly but also began to senesce. While embryos in some pods in the WS treatment that remained green grew at the same rate as those in the WW treatment, ultimately all pods abscised/senesced from plants when FTSW had decreased to ≤0.57, suggesting that pod and seed abortion was induced by either reduced assimilate supply to the developing pod due to stomatal closure and the decrease in leaf photosynthesis, or by ABA accumulation in the seed or pod, or both. The implications of these major findings are discussed.

Previous studies have shown that there are genetic differences in the threshold values of FTSW at which transpiration begins to decrease in chickpea (Zaman-Allah *et al.*, 2011; Pushpavalli *et al.*, 2015), pearl millet (*Pennisetum glaucum* L.) (Kholova *et al.*, 2010), soybean, and peanut (*Arachis hypogaea* L.) (Bhatnagar-Mathur *et al.*, 2007), but this has not been linked to FTSW threshold values at which leaf water potential, photosynthetic rate, and stomatal conductance began to decrease nor with when flower, pod, and seed accumulation cease under terminal drought. While there were no differences in the FTSW threshold values for these parameters between the two chickpea genotypes in the present study, the detailed data on the threshold values for reproductive development in drought-stressed chickpea showed that FTSW values at which pod filling and seed set ceased were much higher than those at which the production of flowers and pods stopped. The FTSW thresholds at which stomatal conductance and seed filling began to decrease in the present study of chickpea were similar to those reported for grass pea, while those at which the photosynthetic rate began to decrease, and flower and pod production ceased, were lower in chickpea than in grass pea (Kong *et al.*, 2015). Thus, it appears that chickpea is better able to maintain flower and pod production under low soil water content than grass pea (Kong *et al.*, 2015), but the flowers and pods that were initiated by chickpea at low soil water contents did not produce seeds.

Reproductive processes, including flower production and pod and seed set, were all reduced under WS. Seed set began to decrease when FTSW fell below 0.57, whereas photosynthesis and therefore presumably assimilate supply only decreased rapidly when FTSW fell below 0.40. Thus, the initial decline in seed set was probably not triggered by a reduction in assimilate supply. Moreover, poor pollen viability and growth in the stigma also do not appear to be the cause of pod and seed abortion. Although the terminal drought reduced pollen viability and pollen germination *in vitro* and the *in vivo* number of pollen tubes in each pistil was decreased in both genotypes at 0.18 FTSW remaining, at least one pollen tube reached the ovary in all cases examined. Further, when FTSW had declined to 0.14, pods were present but many were senescing (yellow in colour) although some were still green; in both cases these small pods had developed from flowers that opened when FTSW was 0.5, which showed that even at severe WS (pre-dawn leaf water potential= –1.5MPa) fertilization occurred in some flowers. Further, in the green pods of the WS plants the embryos continued to develop, at almost the same rate as in the WW plants, for the first 7 d after fertilization. However, both the green and yellow pods in these severely WS plants eventually aborted subsequent to fertilization.

Two explanations are possible for the observed flower and pod abortion in WS plants. First, one cause could be reduced assimilate supply in the flower or developing pod, as found in drought-stressed soybean (Raper and Kramer,1987) and chickpea (Nayyar *et al.*, 2005). In the present study, the stomata began to close at the same soil water content at which seed set was reduced, and the reduction in leaf photosynthesis as the soil dried would limit assimilate availability to the developing pods and seeds. A second cause of abortion could be the ~6-fold higher concentration of ABA in young pods during WS (flowers produced at 0.66 FTSW, pods sampled at 0.18 FTSW) than that for the WW plants. While a proportion of young pods of WS plants (flowers produced at 1.0 FTSW, pods sampled at 0.61 FTSW) and WW plants successfully produced seeds, all young pods of WS plants produced at late stages when FTSW decreased below 0.57 were aborted or senesced, probably resulting from the significantly increased ABA. That increased ABA might also promote flower abortion was implicated by the response of chickpea to cold stress; ABA levels in the female parts (style, ovary) of aborted flowers were 30% and 43% higher than in retained flowers, whereas the male parts (pollen, anthers) did not vary in ABA between the aborted and retained flowers (Nayyar *et al.*, 2005). In addition, increased ABA in young pods might have negative effects on sucrose uptake by seeds, as *in vitro* experiments on wheat grains showed an inhibitory effect of high ABA concentrations on sucrose uptake (Ahmadi and Baker, 1999). The production of ABA by roots in drying soils has been widely reported as a chemical signal to induce stomatal closure (e.g. Davies and Zhang, 1991). In summary for the present experiments, the coincidence of FTSW threshold values for stomatal conductance and seed set, as well as the measured increases in ABA concentrations in the young pods, may imply that ABA induced the closure of stomata leading to a reduction in assimilate production and thus a lower sugar supply to developing seeds, resulting in the cessation of seed set, and/or that ABA might have directly reduced seed set. Although it is difficult to unscramble the direct versus indirect role(s) of ABA, and the possible involvement of other phytohormones, in assimilate production and distribution and direct effects on seed growth, further studies are warranted on detailed mechanisms underlying chickpea flower, seed, and pod retention/abortion and growth and development, and with experiments designed to overcome the challenge to measure the ABA concentrations (and other hormones and metabolites) in various tissues of the reproductive organs just prior to the abortion events.

Mean seed size was also reduced under WS in this study. The rapidly decreased photosynthesis when FTSW fell below 0.40 and therefore presumably restricted assimilate supply may have resulted in the production of smaller seeds and the subsequent abortion of seeds, particularly in the late-formed seeds. The decrease in seed size was greater in DICC8156 (37%) than in DICC8172 (23%), as a result of the difference between DICC8156 and DICC8172 in the final seed size of the early-initiated pods. In DICC8172, the final size of seeds from pods initiated prior to, but developed during, the WS treatment was similar to that for plants in the WW treatment, whereas the corresponding seed size in DICC8156 was ~20% smaller than that in the controls. This suggests that DICC8172 ‘gave preference’ for assimilates to the first seeds developed. The reduction in seed size under terminal drought was consistent with the findings of Davies *et al.* (1999) where terminal drought during seed filling in the field reduced the duration and rate of seed filling, leading to 19–34% reductions in seed size in three chickpea genotypes. The larger seeded genotype in the present study still had larger seeds under drought stress, suggesting that selection for large seeds under favourable conditions would also result in larger seeds under terminal drought.

In summary, our field experiments showed that chickpea genotypes grown under dryland conditions exhibited a 2-fold range in seed yield. When two of these genotypes were subjected to terminal drought from the early podding stage in a glasshouse experiment, the seed yield was reduced by 85% compared with the well-watered plants, as a result of reduced flower, pod, and seed production, increased flower, pod, and seed abortion, and reduced seed size. The imposition of a slow, steady decrease in soil water content revealed that leaf stomatal conductance, leaf transpiration rate, cumulative filled pod number, and cumulative seed number began to decrease when 40% (FTSW ~0.6) of the available water had been transpired, while the pre-dawn leaf water potential and the leaf photosynthetic rate began to decrease when 60% (FTSW ~0.4) of the available soil water had been transpired. Flowers continued to be produced and pods were initiated until 80% (FTSW ~0.2) of the available soil water had been transpired, but these pods failed to develop to maturity. The ABA concentration in the young pods at 9 DAF, sampled at 0.18 FTSW, was ~6-fold greater than that in young pods from WW plants, but these pods and seeds ultimately abscised before maturity of the WS plants. Thus, this study presents data on the ABA concentration in developing pods of chickpea under drought for the first time and shows that the soil water content (i.e. FTSW) at which the production of filled pods and seeds ceased coincided with that at which leaf stomatal conductance and leaf transpiration rate also first decreased, strongly suggesting a role for ABA in the failure to set seeds either directly through abscission of the developing pod or seed, or indirectly through the reduction in assimilate supply to the seed, thereby providing a mechanistic context for the reduced reproductive growth of chickpea during a water deficit.

## Supplementary data

Supplementary data are available at *JXB* online.

Figure S1. Daily rainfall, and daily minimum and maximum air temperatures during the growing season at York, Bindi Bindi, and Cunderdin.

Figure S2. Seed yield of 108 chickpea genotypes in the field at York; and 62 chickpea genotypes at Bindi Bindi and Cunderdin.

Figure S3. Mean volumetric soil water content at different soil depths with time after the start of the water treatments in the water-stressed and well-watered treatments.

Figure S4. Pre-dawn leaf water potential, rate of leaf photosynthesis, stomatal conductance, and rate of leaf transpiration in well-watered and water-stressed treatments in two chickpea genotypes.

Figure S5. Split-line regression between the cumulative number of flowers, total pods, filled pods, and seeds per plant and the fraction of transpirable soil water in the water-stressed treatment in two chickpea genotypes showing break point values where the slope of the fitted regression changed significantly.

Figure S6. Style of a chickpea flower showing pollen on the stigma and pollen tubes in the style; and a pollen tube reaching the ovary of the flower after growing down the style.

Figure S7. Effects of the water treatments on pod development at 16 DAF from flowers produced when the fraction of transpirable soil water of the water-stressed treatment was 0.50 and FTSW at the time of sampling was 0.14.

## Supplementary Material

supplementary_figure_S1_S7
