# Supplementary material for: Response of chickpea (Cicer arietinum L.) to terminal drought: leaf stomatal conductance, pod abscisic acid concentration, and seed set
Source: J Exp Bot. 2016 Apr 20;68(8):1973–85. doi: 10.1093/jxb/erw153 (PMC5429003; doi:10.1093/jxb/erw153)

Response of chickpea (*Cicer arietinum* L.) to terminal drought: leaf stomatal conductance, pod abscisic acid concentration and seed set

Jiayin Pang, Neil C Turner, Tanveer Khan, Yan-Llei Du, Jun-Lan Xiong, Timothy D Colmer, Rosangela Devilla, Katia Stefanova, Kadambot HM Siddique

Supplementary figure captions

Fig. S1. Daily rainfall, and daily minimum and maximum air temperatures during the growing season measured 3 km from the site at York (A) in 2012, 5 km from the site at Bindi Bindi (B), and 4 km from the site at Cunderdin (C) in 2013.

Fig. S2. Seed yield of 108 chickpea genotypes in the field at York (31.89° S, 116.77° E) (A) in 2012; and 62 chickpea genotypes at Bindi Bindi (30.67° S, 116.46° E) (B) and Cunderdin (31.64° S, 117.24° E) (C) in 2013 (means ± s.e., n=3). Columns in red and blue highlight the genotypes DICC8172 and DICC8156, respectively. There was a significant genotype by environment interaction ($P < 0.001$). The $LSD_{0.05}$ values were 239, 241 and 133 kg ha^{-1} for York, Bindi Bindi and Cunderdin, respectively. Seeds were sown at York on 5 June 2012, at Bindi Bindi on 11 May 2013 and at Cunderdin on 28 May 2013 at a depth of 30 mm and a seeding rate of 100 kg ha^{-1}, giving 30–40 plants m^{-2}. Each plot at York was 1.5 m wide (6 rows, 0.25 m apart) and 6 m long, while at Bindi Bindi and Cunderdin plots were 1.5 m wide (6 rows, 0.25 m apart) and 8 m long. Each site had three replicates of each genotype. All seeds were inoculated with a commercial granular chickpea inoculum *Bradyrhizobium* (Nodulator®, Becker-Underwood, Somersby, NSW, Australia) at sowing. Every plot received 100 kg ha^{-1} diammonium phosphate providing 18 kg N ha^{-1} and 20 kg P ha^{-1} drilled with the seeds at sowing. Broad-leaved and grass weeds were controlled by pre-seeding chemical spray (2 L ha^{-1} glyphosate, 1 L ha^{-1} chlorpyrifos, 2 L ha^{-1} trifluralin, 0.9 L ha^{-1} simazine and 0.1 L ha^{-1} isoxaflutole) and hand weeding. Native budworm (*Helicoverpa* spp.) was controlled using 0.3 L ha^{-1} Alpha-Cypermethrin (Fastac® Duo, Nufarm, Victoria, Australia). One L ha^{-1} Chlorothalonil (Bravo®, Syngenta, Waterford, Ireland) was sprayed as a prophylactic spray as required to control ascochyta blight (*Ascochyta rabiei*). Seeds were machined harvested on 24 November 2012 (172 days after sowing (DAS)) at York, 21 November 2013 (194 DAS) at Bindi Bindi and 13 November 2013 (169 DAS) at Cunderdin,

Fig. S3. Mean volumetric soil water content at different soil depths with time after the start of the water treatments (100 DAS) in the water-stressed (A) and well-watered (B) treatments. Data are pooled means ± s.e. (n=3) of the two genotypes and two containers per replicate. Note s.e. values are smaller than the symbols in all cases.

Fig. S4. Change with time after water treatments were imposed on predawn leaf water potential (A), rate of leaf photosynthesis (B), stomatal conductance (C) and rate of leaf transpiration (D) in well-watered (WW) and water-stressed (WS) treatments in two chickpea genotypes, DICC8156 and DICC8172. Data are means ± s.e. (n=3). For predawn leaf water potential, there was a significant interaction of water treatment and time ($P < 0.001$, $LSD_{0.05} = 0.11$ MPa), but not of genotype or three way interaction among genotype × treatment × time. For the rate of photosynthesis, there was a significant effect of genotype ($P < 0.001$, $LSD_{0.05} = 0.9$ μmol m^{-2} s^{-1}) and an interaction of water treatment and time ($P < 0.001$, $LSD_{0.05} = 3.3$ μmol m^{-2} s^{-1}). There was a significant three-way interaction among genotype × water treatment × time for both stomatal conductance ($P < 0.01$, $LSD_{0.05} = 0.03$ mol m^{-2} s^{-1}) and transpiration rate ($P < 0.01$, $LSD_{0.05} = 0.90$ mmol m^{-2} s^{-1}).

Fig. S5. Split-line regression between the cumulative number of flowers, total pods, filled pods, and seeds per plant and the fraction of transpirable soil water in the water-stressed (WS) treatment in two chickpea genotypes, DICC8156 (A) and DICC8172 (B) showing break point values where the slope of the fitted regression changed significantly. Data are means of three replicates.

Fig. S6. Style of a chickpea flower showing pollen (bright coloured grains) on the stigma and pollen tubes (bright lines) in the style (A); and a pollen tube (arrow) reaching the ovary of the flower after growing down the style (B). Scale bar = 100 μm.

Fig. S7. Effects of the water treatment on pod development at 16 days after flowering from flowers tagged when the fraction of transpirable soil water (FTSW) was 0.50 and FTSW at the time of sampling was 0.14. Upper: pods from water-stressed treatment, all pods small and senescing; Lower: pods from well-watered treatment, all pods large and green.

Fig. S1 Pang et al.

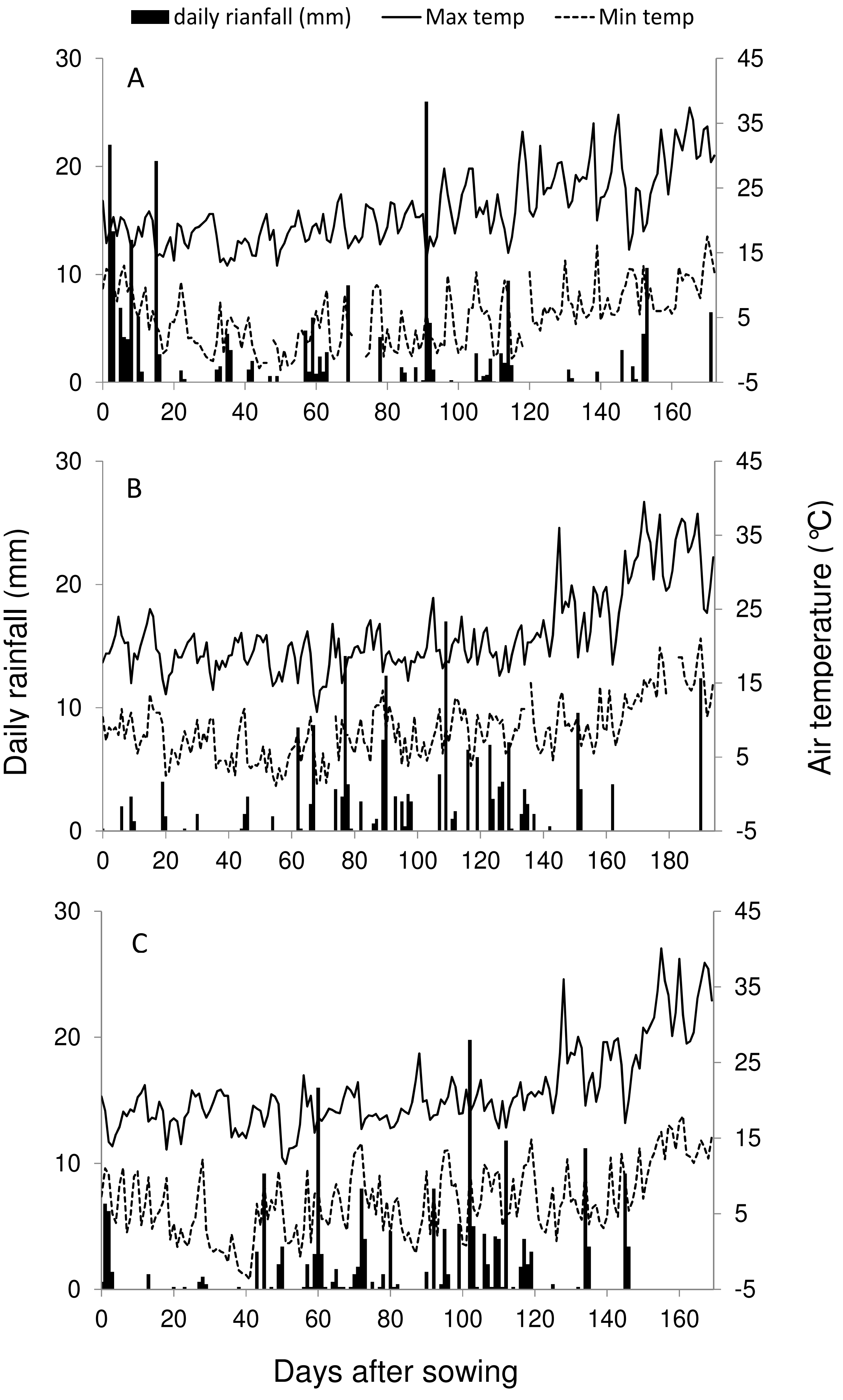

Fig. S2-A Pang et al.

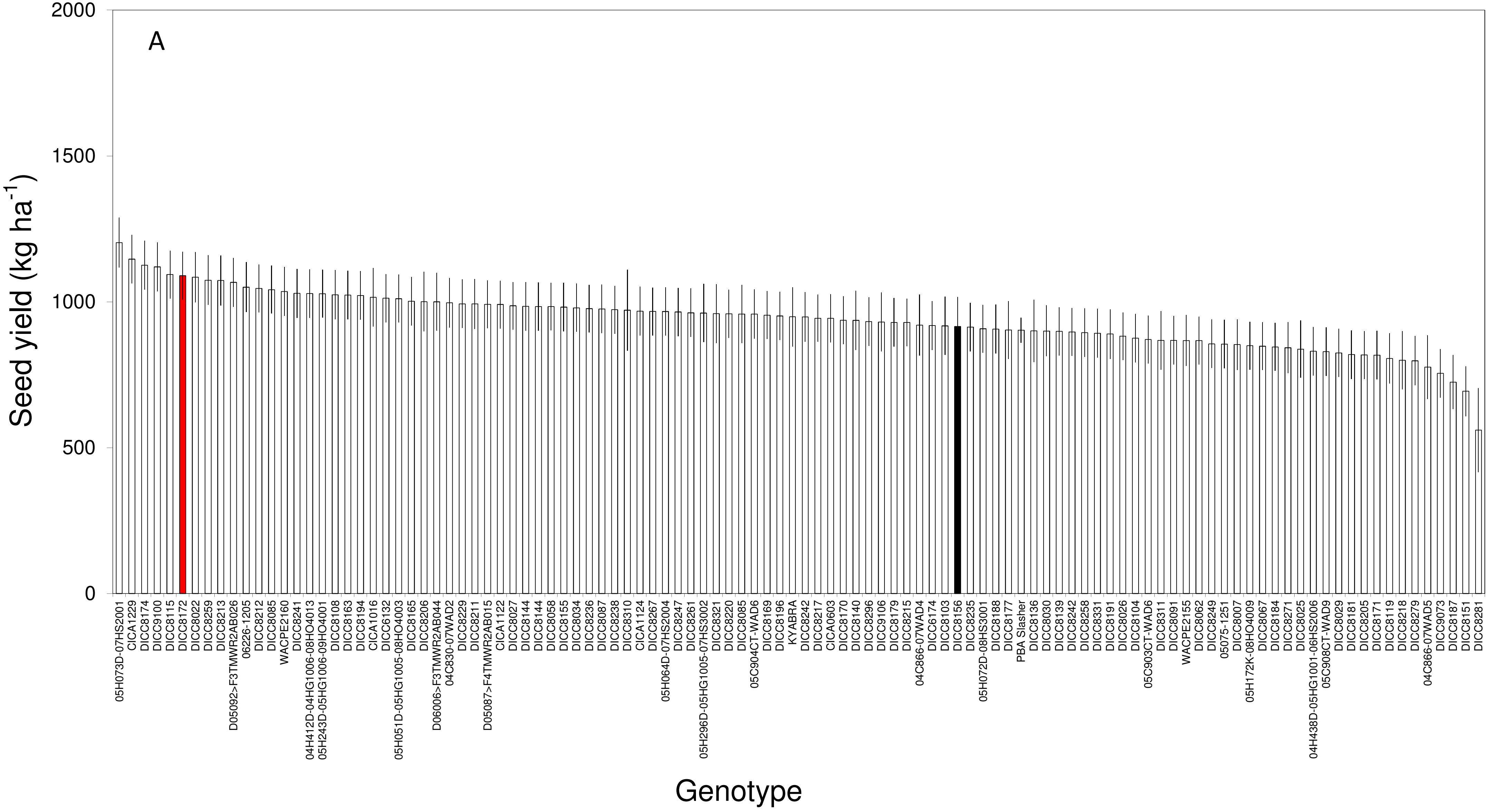

Fig. S2-B Pang et

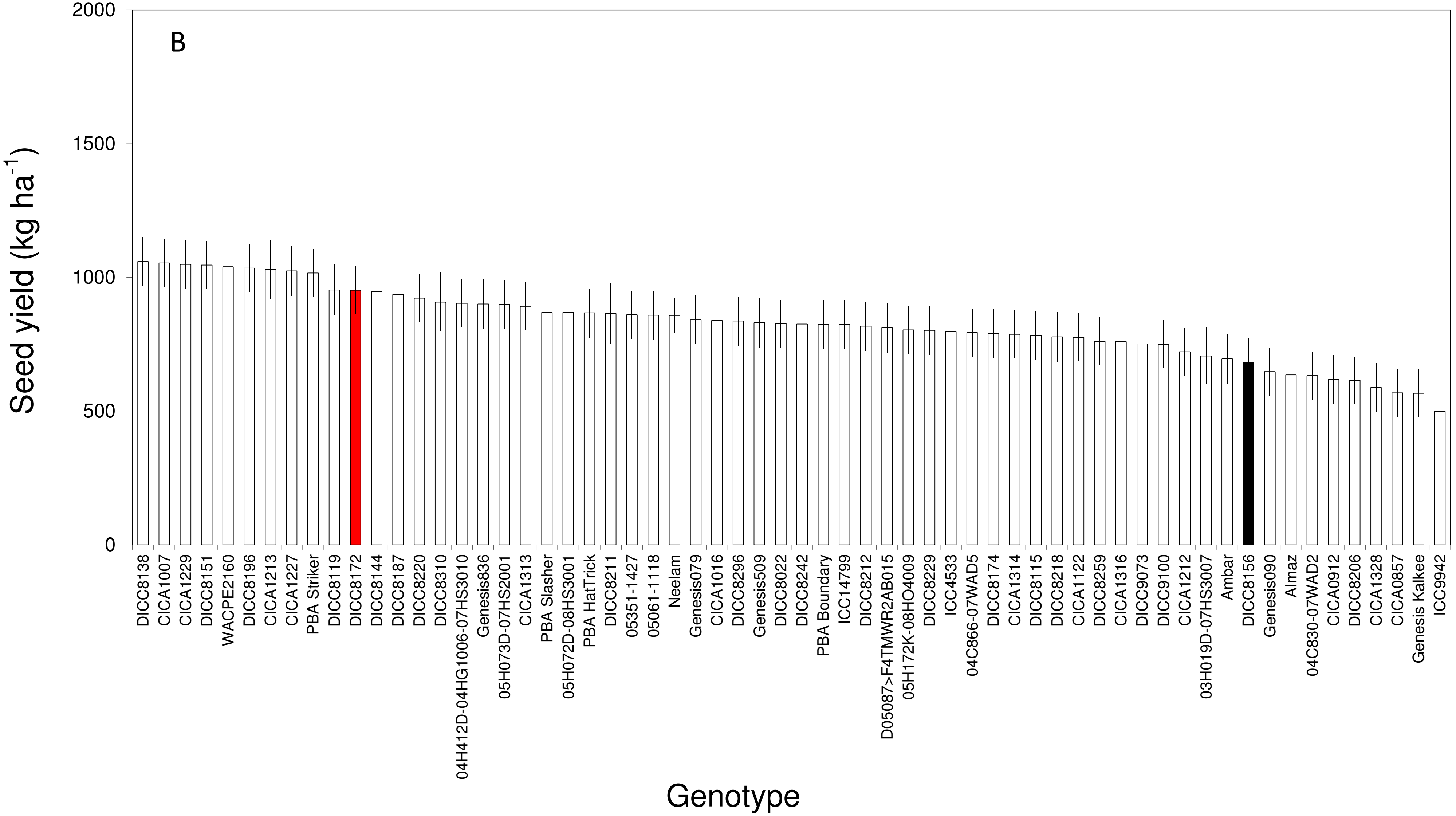

Fig. S2-C Pang et al.

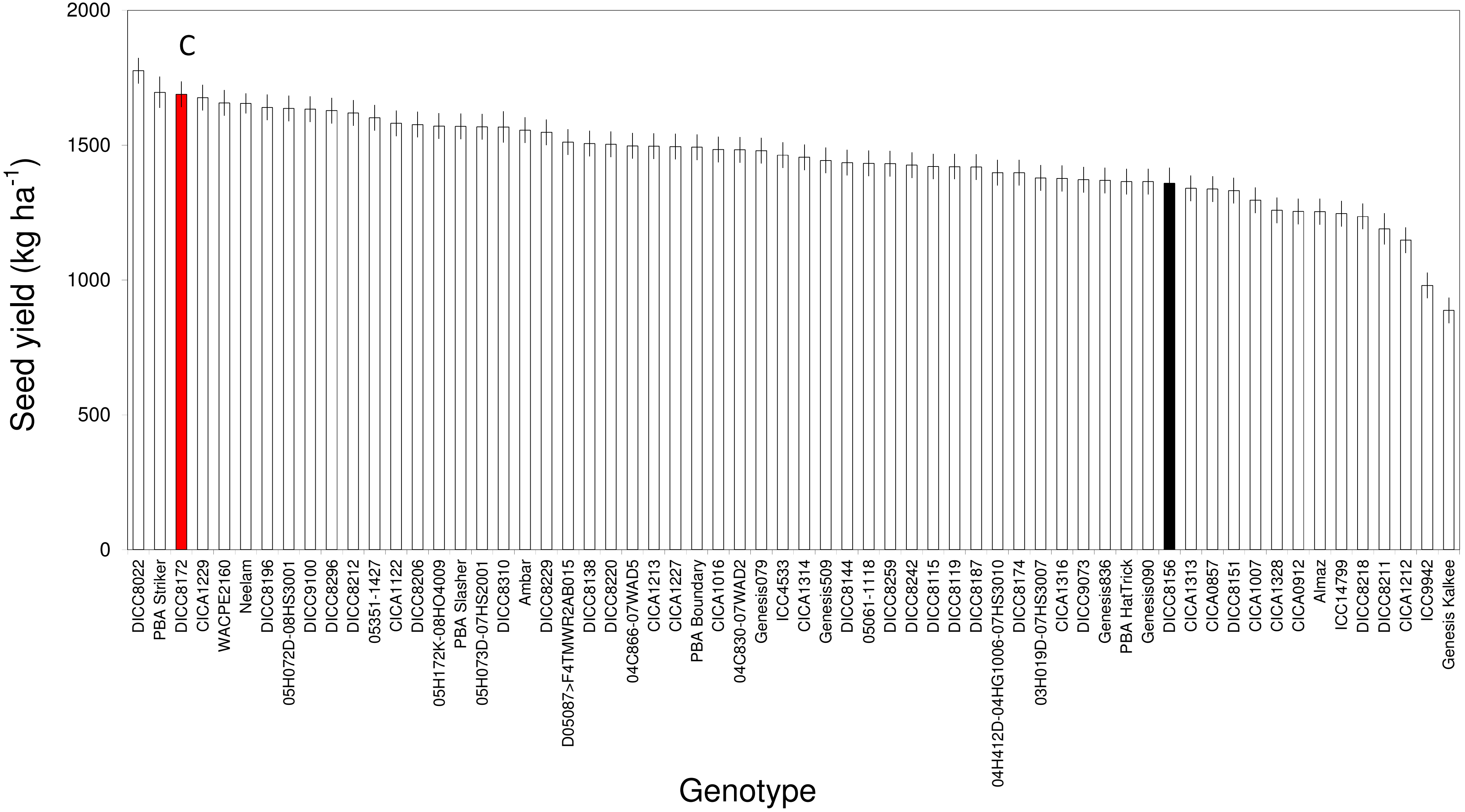

Fig. S3 Pang et al.

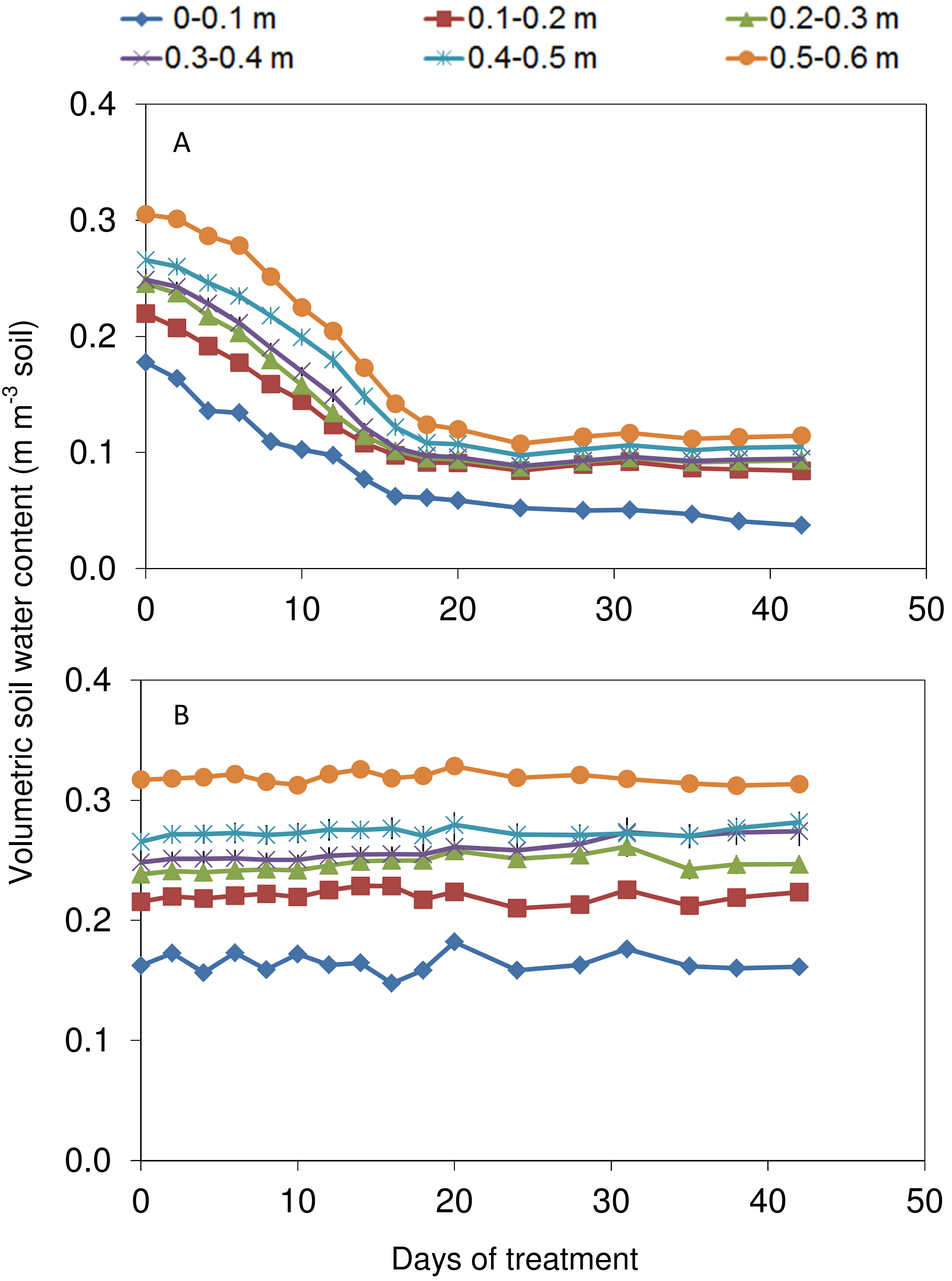

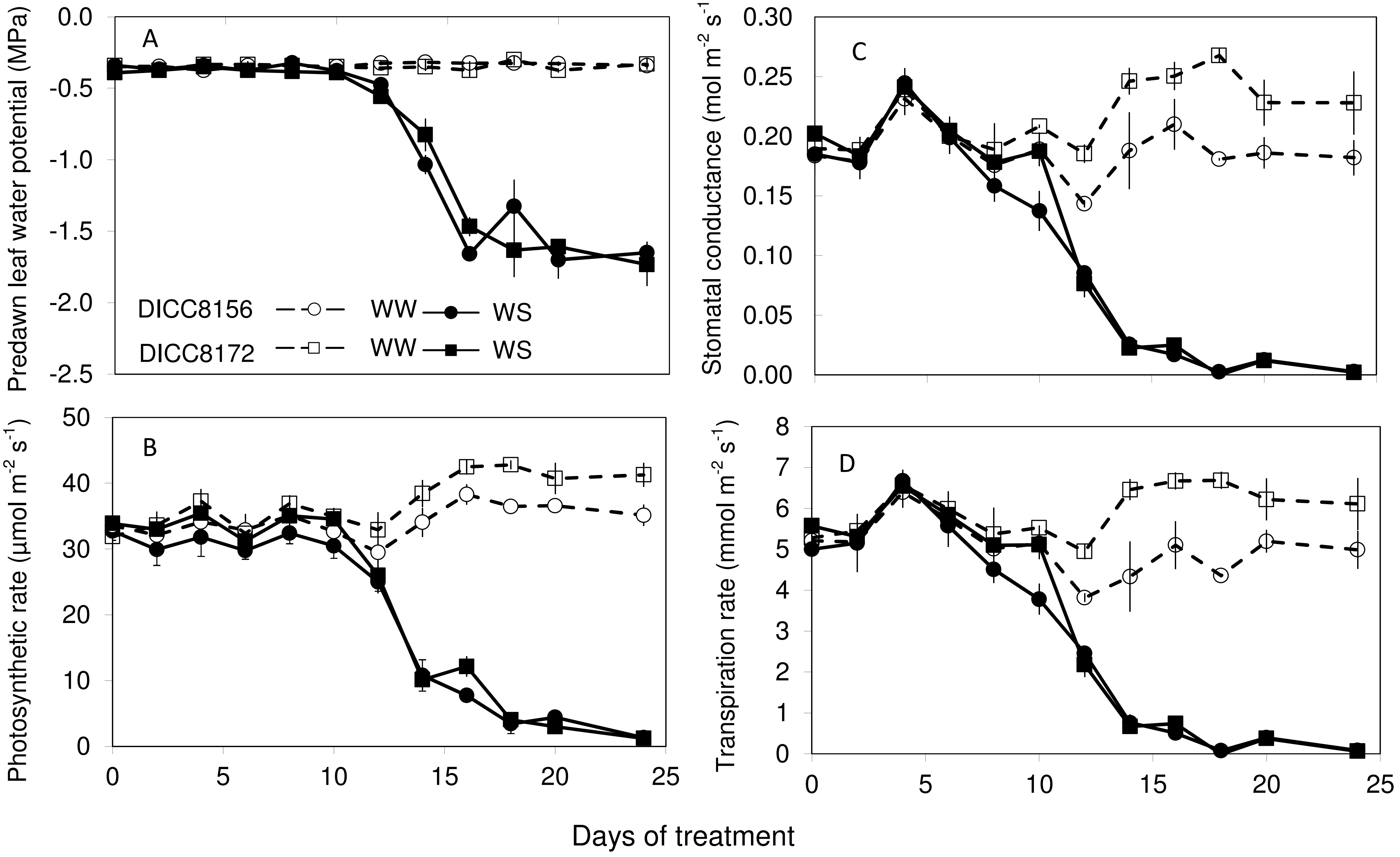
A
B
C
D
Predawn leaf water potential (MPa)
Photosynthetic rate (μmol m-2 s-1)
Stomatal conductance (mol m-2 s-1)
Transpiration rate (mmol m-2 s-1)
DICC8156
DICC8172
WW
WS
Days of treatment

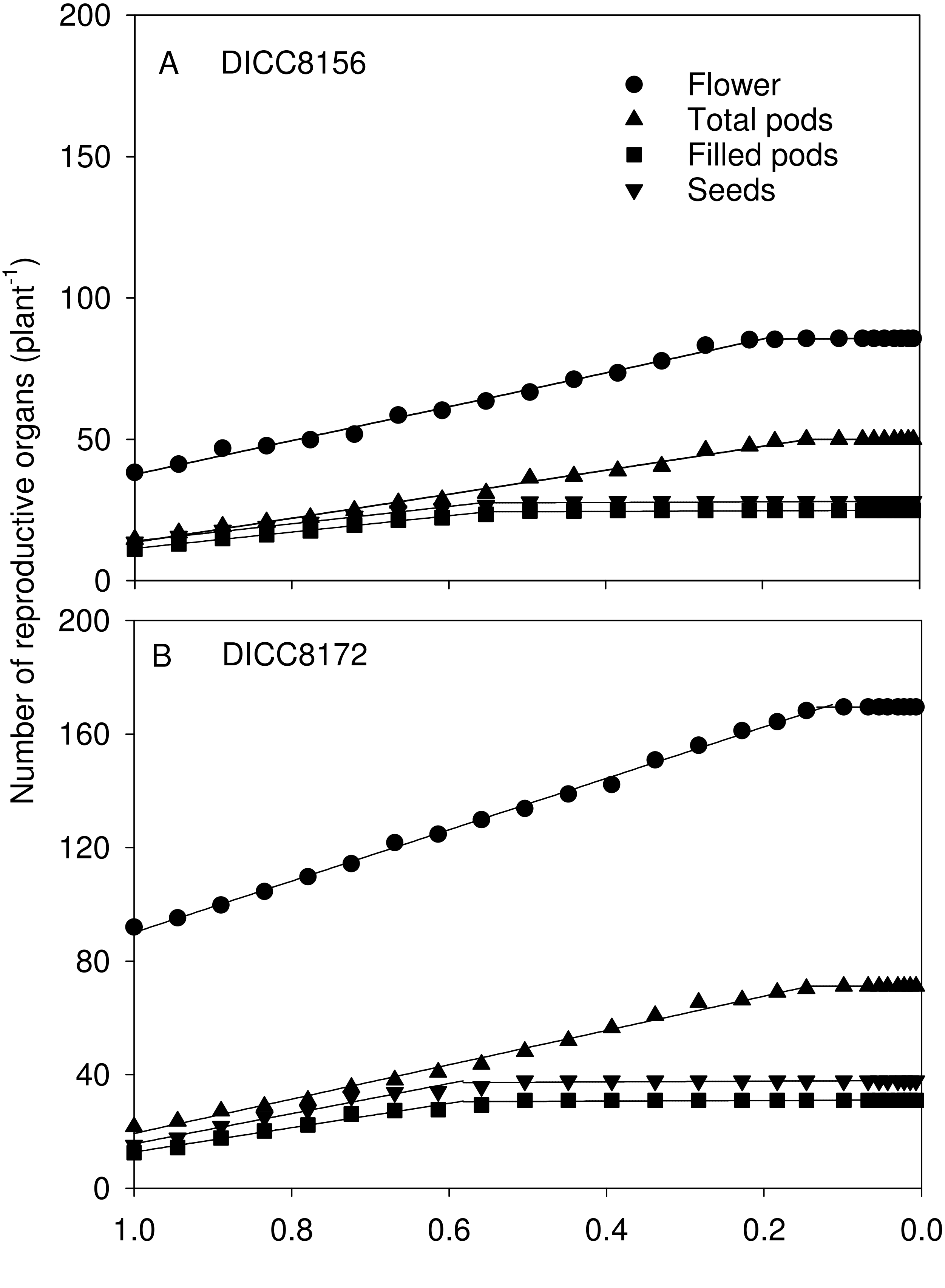

A DICC8156
Flower
Total pods
Filled pods
Seeds
B DICC8172
Number of reproductive organs (plant⁻¹)
Fraction of transpirable soil water
200
150
100
50
0
200
160
120
80
40
0
1.0
0.8
0.6
0.4
0.2
0.0

Fig. S6 Pang et al.

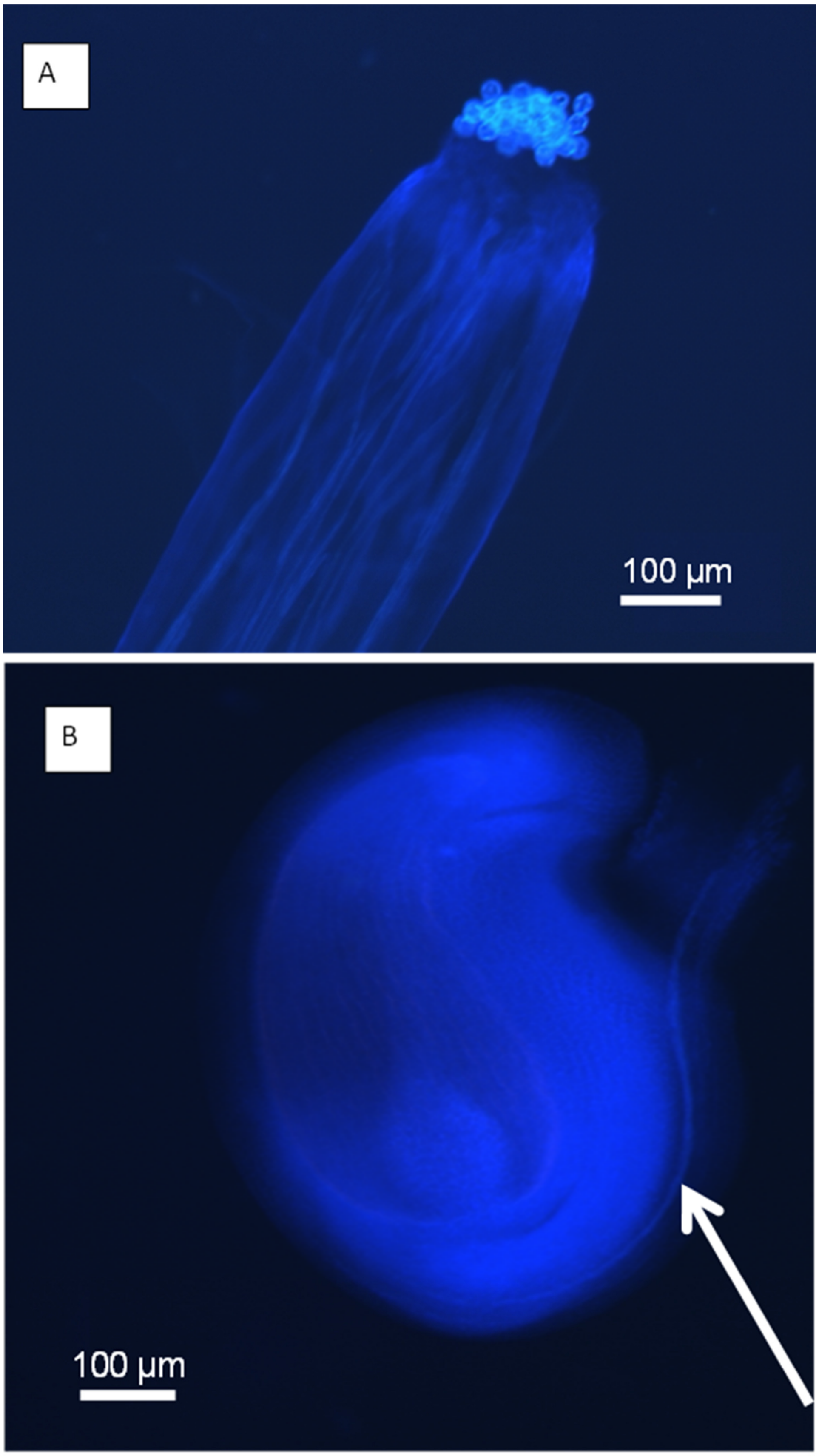

Fig. S7 Pang et al.

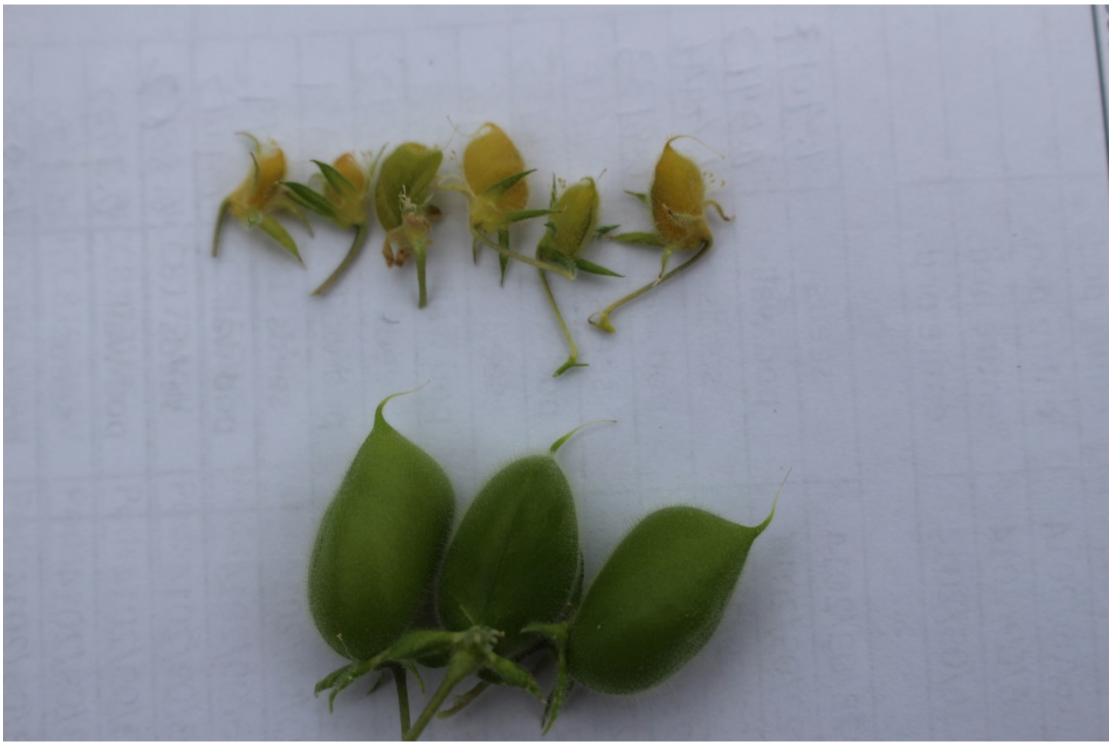

Supplement: supplementary_figure_S1_S7 [file erw153_suppl_supplementary_figure_S1_S7.pdf]
